# Mood disorders in Huntington's disease: from behavior to cellular and molecular mechanisms

**DOI:** 10.3389/fnbeh.2014.00135

**Published:** 2014-04-23

**Authors:** Patrick Pla, Sophie Orvoen, Frédéric Saudou, Denis J. David, Sandrine Humbert

**Affiliations:** ^1^Institut CurieOrsay, France; ^2^CNRS UMR3306Orsay, France; ^3^INSERM U1005Orsay, France; ^4^Faculté des Sciences, Université Paris-SudOrsay, France; ^5^EA3544, Faculté de Pharmacie, Université Paris-SudChâtenay-Malabry, France

**Keywords:** Huntington's disease, depression, anxiety, neurogenesis, Huntingtin, BDNF, serotonin, HPA axis

## Abstract

Huntington's disease (HD) is a neurodegenerative disorder that is best known for its effect on motor control. Mood disturbances such as depression, anxiety, and irritability also have a high prevalence in patients with HD, and often start before the onset of motor symptoms. Various rodent models of HD recapitulate the anxiety/depressive behavior seen in patients. HD is caused by an expanded polyglutamine stretch in the N-terminal part of a 350 kDa protein called huntingtin (HTT). HTT is ubiquitously expressed and is implicated in several cellular functions including control of transcription, vesicular trafficking, ciliogenesis, and mitosis. This review summarizes progress in efforts to understand the cellular and molecular mechanisms underlying behavioral disorders in patients with HD. Dysfunctional HTT affects cellular pathways that are involved in mood disorders or in the response to antidepressants, including BDNF/TrkB and serotonergic signaling. Moreover, HTT affects adult hippocampal neurogenesis, a physiological phenomenon that is implicated in some of the behavioral effects of antidepressants and is linked to the control of anxiety. These findings are consistent with the emerging role of wild-type HTT as a crucial component of neuronal development and physiology. Thus, the pathogenic polyQ expansion in HTT could lead to mood disorders not only by the gain of a new toxic function but also by the perturbation of its normal function.

## Introduction

Huntington's disease (HD) is a fatal neurodegenerative disorder characterized by cognitive and psychiatric disturbances that are associated with loss of motor control, including chorea, dystonia, and a lack of gestural coordination (Novak and Tabrizi, 2011). These symptoms generally start to appear in midlife. A broad range of cognitive disturbances appear during HD progression. Early cognitive defects are characterized by poor adaptation and planification associated with deficits in executive functioning and perseveration. Other defects are most prevalent during the late stages of the disease, like learning difficulties and working and long term memory deficits, leading to dementia (Rosenblatt, 2007; Paulsen, 2011).

HD is caused by an abnormal expansion of glutamine (polyQ) in the N-terminal part of the 350 kDa protein huntingtin (HTT). This stretch is encoded by a trinucleotide CAG repetition in exon 1 of *HTT*. An expansion greater than 36 repeats results in HD (Huntington Collaborative Research Group, 1993). The number of repeats is inversely correlated with the age of onset of motor symptoms and disease onset during childhood or adolescence is associated with more than 60 CAG repeats (Walker, 2007). HD is inherited in an autosomal dominant manner. Genetic and molecular studies have suggested that mutated polyQ-HTT leads to neuronal damage by gain of toxic function. The pathogenic mechanism requires the cleavage of full-length HTT into smaller N-terminal fragments that contain the polyQ stretch that have been shown to be highly toxic in HD cellular and mouse models (Gafni et al., 2004; Graham et al., 2006). However, there is strong evidence that loss of the normal functions of wild-type HTT also plays a role in pathological mechanisms of HD.

## Mood disorders in patients with HD

Mood disturbances are prevalent not only among patients with diagnosed HD but also in pre-symptomatic carriers of the HD gene (Duff et al., 2007; Julien et al., 2007; Rosenblatt, 2007) (Table 1). These disturbances include impulsivity, irritability (which can be accompanied by aggressiveness), anxiety, and depression. Major depression is the most common symptom among pre-symptomatic HD carriers, although some patients will have only part of the symptoms of the major depression, or limited in intensity or time (Epping and Paulsen, 2011; Reedeker et al., 2012). Maniac depressive disorder is not particularly prevalent in HD (Van Duijn et al., 2008; Epping and Paulsen, 2011). Mood disturbances may precede the onset of the motor phenotype by 4–10 years, making them one of the earliest symptoms of HD. The severity of depression in HD is not dependent on disease progression (Berrios et al., 2001; Craufurd et al., 2001; Kingma et al., 2008). This suggests that different mechanisms affect neurons involved in mood regulation and neurons involved in motor and cognitive skills that are disabled in late phases of the disease in HD patients. However, mood, and cognitive alterations may also share some pathogenic mechanisms as some aspects of these alterations are found associated in the general population and in HD patients. In particular, depression and memory impairments are observed together and this is also the case for cognitive flexibility and planification defects (Smith et al., 2012; Trivedi and Greer, 2014). Although apathy is part of symptoms of major depression, frequency, and intensity of apathy in HD patients increase with disease progression contrary to depression (Kingma et al., 2008; Van Duijn et al., 2010). Thus, as previously suggested (Levy et al., 1998), apathy appears not only as a symptom of depression but as a syndrome with its own dynamics and mechanisms.

**Table 1:** **Clinical observations on Huntington's disease patients**.

**Clinical studies (number of patients)**	**Psychiatric observations**	**Scale used to assess depression and/or anxiety**	**Use of pharmacologic treatments**	**References**
217 patients with motor symptoms (comparison black vs. white patients)	32.8% had affective disorders Age at onset of motor symptoms for persons with affective disorder was significantly later than that for persons without affective disorder (43.4 and 38.7 years, respectively).	DSM-III criteria Diagnostic Interview Schedule	ND	Folstein et al., 1987
134 patients Male subjects = 47%	39% were depressed 16% had attempted suicide 34% had suicidal ideation	UHDRS PBA-HD	34% were given antidepressant drugs. 24% were given sedative or anxiolytic treatment	Craufurd et al., 2001
2835 patients Male subjects = 47.5%	More than 40% of patients have depressive symptoms. More than 10% have attempted suicide	UHDRS	More than 50% had received antidepressant treatments in the past	Paulsen et al., 2005
681 patients Male subjects = 46%	More psychiatric symptoms (e.g., depression, anxiety, obsessive-compulsiveness) than healthy people. Higher levels of psychiatric symptoms in individuals with severe motor impairment	UHDRS SCL-90-R	ND	Duff et al., 2007
204 participants, blind to their genetic status, asymptomatic (definite HD were excluded)	20% had experienced major depression 17% had an anxiety disorder, and 11% had general anxiety. The rate of depression increased as a function of proximity to clinical onset	CIDI DSM-III criteria	ND	Julien et al., 2007
254 at risk of HD or recently diagnosed participants Male subjects = 29%	Increased anxiety score in presymptomatic carriers and patients with HD individuals. ≈25% of presymptomatic carriers and patients with HD were depressed Prevalence of symptoms increases with the progression of HD.	SCL-90-R CES-D	21% were given antidepressant, anxiolytic or antipsychotic treatments	Marshall et al., 2007
152 patients, including pre-symptomatic Male subjects = 45.5%	Higher prevalence of depression in HD patients, including pre-symptomatic. No higher prevalence with development of HD	PBA-HD	ND	Kingma et al., 2008
154 patients including pre-symptomatic carriers Male subjects = 45.7%	Higher prevalence of depression/anxiety in presymptomatic and symptomatic HD patients than average population.	DSM-IV criteria CIDI	26.4% were given antidepressant treatment	Van Duijn et al., 2008
111 patients	Higher prevalence of neuropsychiatric symptoms in patients with HD than average population. Prevalence did not increase with the progression of HD.	PBA-HD	ND	Thompson et al., 2012
803 prodromal patients with HD mutation Male subjects = 36%	Depression prevalence higher in females.	BDI-II SCL-90-R UHDRS	ND	Epping et al., 2013

*BDI-II, Beck Depression Inventory; CES-D, Center for Epidemiological Studies Depression Scale; CIDI, Composite International Diagnostic Interview; DSM-III/IV, Diagnostic and Statistical Manual of Mental Disorders 3rd/4th edition; PBA-HD, Problem Behaviors Assessment for Huntington Disease; SCL-90-R, Symptom Checklist 90 Revised; UHDRS, Unified Huntington Disease Rating Scale; ND, no data*.

The reasons underlying the frequent co-morbidity of HD and mood disorder are still unclear. Family history of HD, especially given that HD shows a dominant mode of inheritance, is probably an important factor. Indeed, an individual's knowledge that he or she is a carrier and will inevitably experience neurological symptoms, could contribute to the prevalence of psychiatric disturbances. However, this explanation alone is not sufficient to explain the high prevalence of psychiatric disturbances in HD (Almqvist et al., 2003; Julien et al., 2007; Van Duijn et al., 2008). Therefore, there are probably one or several molecular mechanisms, independent of the psychosocial environment, that can explain the prevalence of mood disorders in patients or carriers of HD. This hypothesis can be tested in animal models of HD.

There is no treatment that can halt or even slow down the progression of Huntington's disease. Current effective treatments aim to ease the symptoms of this disease. The evidence base for drugs in HD is very small so the choice of pharmacological agents is based mainly on clinical experience. Various molecules are used to treat motor dysfunction. These include neuroleptics for chorea, and tetrabenazine (which is a dopamine-depleting agent that inhibits the vesicular dopamine transporter VMAT2) for dyskinesia. Tetrabenazine is the most effective drug for the management of HD and hence is the first choice of treatment; however, this drug induces depression and sedation in patients (Frank, 2010; Mestre and Ferreira, 2012) and similar findings were reported in a mouse model of HD (YAC128) (Wang et al., 2010). Stress, anxiety, and depression can aggravate chorea, so measures to treat these mood problems may also help to slow the progression of HD. Mood disorders in HD are treated by antidepressant and/or anxiolytic drugs (Table 1). Pre-symptomatic HD carriers are almost twice as likely to be treated with antidepressants than individuals who are not carriers of HD (Rowe et al., 2012). At present, there is no established molecular evidence base for the treatment of depression in HD. However, antidepressants are often very effective in clinical practice (Novak and Tabrizi, 2011). Depression is most commonly treated with classic serotonin-specific reuptake inhibitors (SSRIs) such as citalopram or sertraline. Fluoxetine is often avoided as it exacerbates chorea and enhances anxiety, both of which frequently occur in patients with HD (Chari et al., 2003; Novak and Tabrizi, 2011). Anxiety is treated with non-stimulating SSRIs such as buspirone or benzodiazepines (Novak and Tabrizi, 2011).

## Mouse models of HD

Various rodent models have been used to study the effect of polyQ expansion in HTT (Heng et al., 2008; Pouladi et al., 2013). These models differ in the number of CAG repeats, the size of the expressed HTT fragment, and the gene promoter used. Thus, the amount of mutant HTT that is expressed varies between different models. The genetic background of the mouse strains used to design models of HD is also important, especially because various strains perform differently in behavioral tests (Miller et al., 2010; Mozhui et al., 2010). These models can be grouped according to their mode of expression of mutant HTT: transgenic models expressing truncated HTT, transgenic models expressing full-length HTT, and knock-in (KI) models.

The short lifespan of mouse models prevents them from developing the same neuropathological symptoms that develop during decades in human. Large numbers of glutamines in the polyQ stretch in the homozygous state in KI models and/or a high abundance of transgenic mutant proteins are necessary to obtain a robust phenotype. The length of the N-terminal fragments and their toxicity are inversely correlated, which underlies the robust phenotype of models in which a short N-terminal polyQ-HTT fragment is expressed (Hackam et al., 1998; Landles et al., 2010).

The sequence coding the 350 kDa HTT protein contains 67 exons spanning over 170 kb of the mouse genome. Hence, the creation of transgenic models in which full-length HTT is expressed is a difficult task. Therefore in some models, the exogenous protein is limited to the N-terminal part containing the polyQ expansion, with the assumption that this portion is sufficient to recapitulate some aspects of the disease. R6/1 and R6/2 mice carry a transgene encompassing exon 1 of *Htt* (Mangiarini et al., 1996). In R6/1 mice this exon contains a polyQ tract of approximately 115 residues whereas in R6/2 mice this number is 150. The progression of HD in R6/2 and R6/1 mice is particularly fast and aggressive, therefore these mice may only be suitable as models for juvenile HD, which manifests in patients with very extensive polyQ stretches. These models could also correspond to the late HD stages when HTT is cleaved and N-terminal fragments accumulate. N171-82Q mice carry a transgene encompassing the first 171 amino acids of HTT with a polyQ tract of 82 residues (Schilling et al., 1999).

Transgenic models expressing full-length mutant HTT contain the human *HTT* gene with an expanded CAG repeat which is randomly inserted into the mouse genome through a Yeast Artificial Chromosome (YAC) or a Bacterial Artificial Chromosome (BAC). The YAC72 model contains full length *HTT* including 72 CAG repeats and the YAC128 model contains full length *HTT* including 128 CAG repeats (Hodgson et al., 1999). Transgenic *HTT* in BACHD mice contains 97 CAG repeats coding for glutamine (Gray et al., 2008). In these models, transgenic *HTT* is under the control of the human *HTT* promoter. The endogenous wild-type form of HTT is still expressed in these models, which is also the case for models expressing a truncated form of HTT. Interestingly, mouse models expressing full-length mutant HTT generally develop motor deficits later than transgenic models with truncated HTT, which allows more time to study anxio-depressive behaviors without interference from motor impairment in these mice.

KI models are constructed by replacing the murine exon 1 of the endogenous *Htt* gene by a chimeric human/mouse sequence that includes various lengths of the CAG stretch (Menalled et al., 2009). Heterozygous KI HD mice thus mimic the genetic situation of human patients. KI models are also considered a more accurate genetic HD model than many transgenic models, because they express the mutated gene under the control of the endogenous mouse promoter. Thus, the mutated HTT is expressed at levels similar to the endogenous gene, and is not overexpressed as in transgenic models. This may explain why KI mice have a milder phenotype than that of transgenic mouse models. Indeed, KI strains present very little or subtle observable motor dysfunction, and a normal lifespan (Menalled et al., 2009). KI mouse models can be particularly useful to study the early symptoms of the disease, including anxio-depressive disorders, prior to the onset of motor impairments.

Few models have been developed to study the role of wild-type HTT. The knock-out of *Htt* is lethal early in development at embryonic day 7.5 (Duyao et al., 1995; Nasir et al., 1995; Zeitlin et al., 1995); therefore, Cre-Lox systems have been used to study the role of HTT in the developing nervous system or in the adult nervous system (Dragatsis et al., 2000; Dietrich et al., 2009; Pla et al., 2013). *CaMKCreER^*T*2^*; *Htt^flox/flox^* mice were generated to study the function of HTT in mature cortical and hippocampal neurons of adult mice. Following tamoxifen injection (in 2 month old mice), *Htt^flox/flox^* is excised specifically from the genome of these neuronal cells, hence allowing the study of HTT function in adult mice without developmental bias (Pla et al., 2013). KI mice containing point mutations in *Htt* have also been produced to study the role of post-translational modifications of HTT. These models involve modifications of serines 1181 and 1201 that are phosphorylated by Cdk5: mutations either mimic constitutive phosphorylation or prevent phosphorylation at these two sites (Ben M'Barek et al., 2013).

## Mouse models of HD present anxiety/depressive-like phenotypes

The anxio-depressive status of various mouse models of HD has been investigated by classical behavioral tests. Results are summarized in Table 2.

**Table 2:**
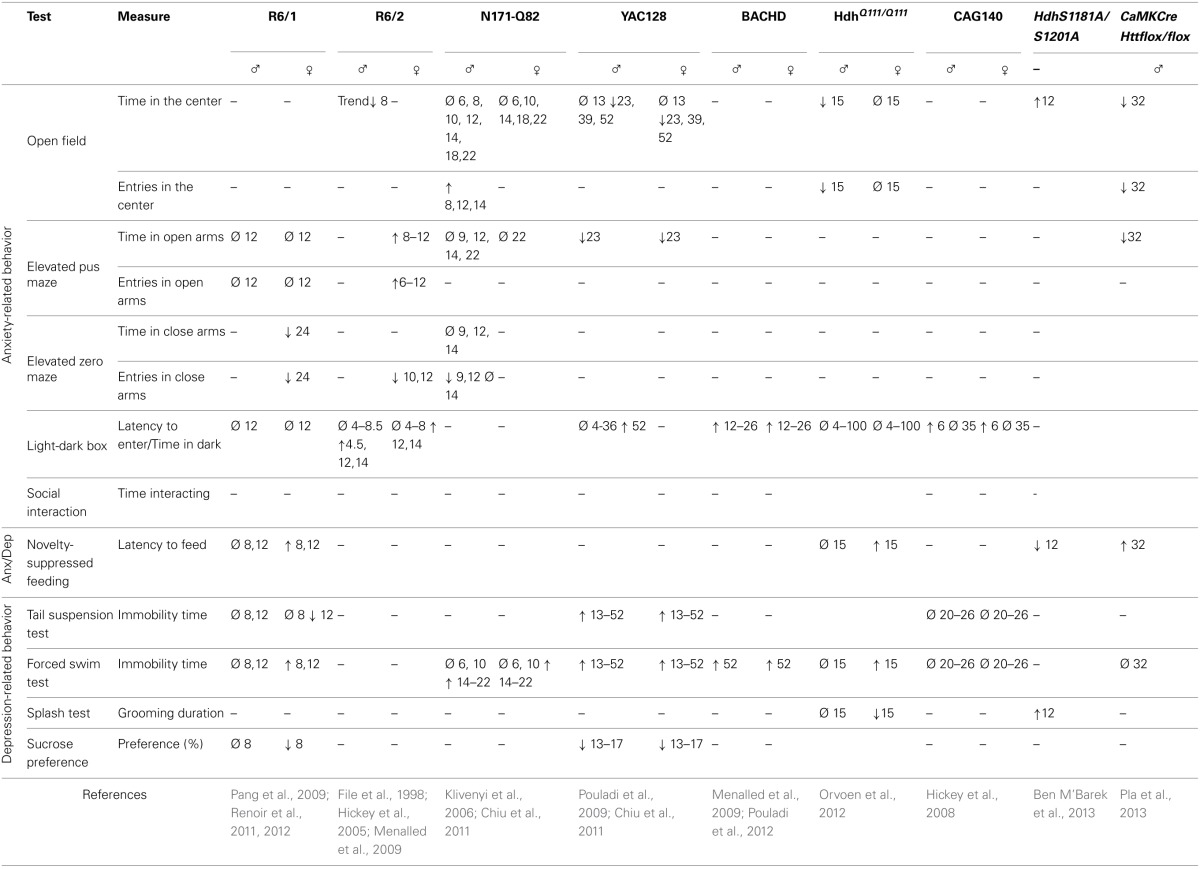
**Anxiety and depression-related behavior in HD murine models**.

*Numbers indicate the age in weeks at which the phenotype was observed. Ø, no significant change; ↑, significant increase; ↓, significant decrease*.

### Anxiety paradigms

The open-field test is frequently used to assess motor activity but can also be applied to measure anxiety. During this test, mice are allowed to explore a walled enclosure for about 30 min. Mice usually spend most of their time along the walls rather than in the middle of the field, because they fear that exposure may make them vulnerable to predators. Therefore, anxiety is inversely correlated with the amount of time the mouse spends in the center, and/or the number of trips it makes from the walls to the center. It is however necessary to verify that the behavioral differences between wild-type and HD mice in this test are not due to an impaired motility of the HD animals. This is particularly important because HD models (and especially the transgenic ones) develop impairment to motor behavior. With this in mind, the most pertinent variable to measure is the “ambulatory distance/total ambulatory distance” ratio. According to this variable, male *Hdh ^Q111/Q111^* mice and *CaMKCreER^*T*2^*; *Htt^flox/flox^* mice show an anxiety-like behavior (Orvoen et al., 2012; Pla et al., 2013). R6/2 mice and YAC128 mice also spend little time in the center of the area (Ciamei and Morton, 2008; Chiu et al., 2011). However, this finding may not be significant for YAC128 mice because they also travel short distances.

The elevated plus maze (EPM) test is based on the aversion of mice to open spaces and height, and is widely-used in the study of anxiety behaviors. In R6/1 mice, anxiety-like behavior appears progressively and reaches its peak in 24 week-old mice. However, this test is difficult to interpret at this age because locomotor activity is also altered. This impairment is shown by the small number of entries that the mouse makes into both the open and closed arms of the maze (Naver et al., 2003; Nithianantharajah et al., 2008). An anxiety phenotype is observed in YAC128 mice, which spend less time in the open quadrants of the zero-maze than in the closed quadrants (Chiu et al., 2011). An anxiety-like behavior is also observed in *CaMKCreER^*T*2^*; *Htt^flox/flox^* mice (Pla et al., 2013), showing that a loss of function of HTT only in mature cortical and hippocampal neurons in adults is sufficient to lead to anxiety.

The light/dark box test is used to assess anxiety-like behaviors and is based on the aversion of mice to light. Variables that are measured include the latency to enter the lit compartment, and the time spent in each compartment. Both male and female R6/2 mice exhibit an anxiety-like behavior from 12 to 14 weeks as assessed by this test. Anxiety may start earlier in males than in females, although this is not a consistent finding (Hickey et al., 2005; Menalled et al., 2009). Menalled and colleagues also showed that 12 week-old BACHD mice spend a long time in the dark box, indicative of an anxiety-like phenotype (Menalled et al., 2009). CAG140 mice show a high latency to enter the lit compartment at 1.5 months of age but not at 8 months of age (Hickey et al., 2008). This suggests that an early anxiety-like phenotype in both males and females disappears as the disease progresses. *Hdh^Q111/Q111^* or YAC128 mice display no phenotype in this test (Chiu et al., 2011; Orvoen et al., 2012).

### Anxiety-depressive paradigms

The novelty suppressed feeding (NSF) test is a conflict test with competing motivations: the drive to eat and the fear of venturing into the center of a brightly lit area. This test measures both anxiety and depression and the outcome of this test is sensitive to anxiolytics and chronic antidepressants, but not to acute antidepressants (David et al., 2009). The NSF test is also described as a “neurogenesis-dependent” test, because the ablation of neurogenesis in the hippocampus blocks the efficacy of antidepressants in this test (David et al., 2009). R6/1, *Hdh^Q111/Q111^*, and *CaMKCreER^*T*2^*; *Htt^flox/flox^* mice show a high latency to eat in the NSF paradigm, suggestive of anxiodepressive-like behavior in these mice (Renoir et al., 2011; Orvoen et al., 2012; Pla et al., 2013).

### Depressive paradigms

The sucrose preference test is used to measure anhedonia, which is a component of depressive behavior in both humans and mice. Studies show that HD mice have a lower preference for sucrose compared to wild-type mice, which represents a depressive-like behavior (Pouladi et al., 2009; Renoir et al., 2011). This behavior is sex-independent in YAC128 mice; however, the saccharin-preference of R6/1 female mice is lower than that of R6/1 male mice, or that of wild-type males or females.

In the splash test, low grooming frequency after a sucrose solution is squirted onto the mouse's fur can be interpreted as a loss of motivational behavior. This is thought to model some symptoms of depression, such as apathic behavior. Female, but not male *Hdh^Q111/Q111^* mice, show a depressive behavior in this test (Orvoen et al., 2012).

The forced swim test (FST) is widely used in pharmacology to screen for molecules with anti-depressant effects. It is also used to highlight a depressive-like behavior in rodents, linked to resignation, although the validity of this test as a measure of depression is debatable. Apart from CAG140, all of the HD models that were subjected to this test show a long duration of immobility, which indicates a depressive-like behavior in this paradigm (Grote et al., 2005; Hickey et al., 2008; Peng et al., 2008; Pouladi et al., 2009; Chiu et al., 2011; Renoir et al., 2011; Orvoen et al., 2012; Pouladi et al., 2012; Renoir et al., 2012). A long duration of immobility is associated with either a short swimming duration (YAC 128) (Pouladi et al., 2009), or a short climbing duration (*Hdh^Q111/Q111^*) (Orvoen et al., 2012). According to Detke et al. (1995) and Page et al. (1999), short swimming duration indicates an alteration in serotonergic circuitry and short climbing duration indicates an alteration in noradrenergic circuitry. In the YAC128 mouse model the severity of depressive-like phenotypes tested with FST does not increase with age or with the progression of motor symptoms (Pouladi et al., 2009). Furthermore, the severity of the depressive-like phenotype is not correlated with the number of CAG repeats (Pouladi et al., 2009), similar to the situation in HD patients (Epping et al., 2013). There appears to be a sex-specific component of this depressive-like phenotype. Indeed, some studies report that mutated *HTT* is associated with a depressive-like phenotype in females but not in males, and in the R6/1 model, this phenotype manifests earlier in females than in males (Grote et al., 2005; Pang et al., 2009; Renoir et al., 2011; Orvoen et al., 2012). Thus, in the FST, the depressive-like phenotype has a sex-specific component, similar to findings for the sucrose-preference test.

The tail suspension test (TST) is another widely-used test in the screening for antidepressant drugs, and is also used to measure depressive-like behavior, linked to resignation, which is a function of the amount of time that the animal stays immobile. YAC128 mice show a high degree of immobility in the TST, which is consistent with findings of the FST (Chiu et al., 2011). However, the findings of the FST and TST involving R6/1 mice are inconsistent: in the TST, the duration of immobility is short for female R6/1 mice aged 12 weeks, suggesting that mutated HTT actually has an “antidepressant” effect (Pang et al., 2009). Nonetheless, another study found that the HD mutation does not affect TST performance of R6/1 mice at 8 weeks of age compared to wild-type mice (Renoir et al., 2011). The immobility duration of male or female CAG140 mice does not differ from that of wild-type mice in the TST, which is consistent with FST results in this strain (Hickey et al., 2008).

Altogether, results from different mouse strains using various behavioral tests show that HD mice exhibit anxiety- and depressive-like behavior. In various mouse models, a sex-specific component to these behaviors is evident: HD mutant females are more prone to depressive-like behaviors than mutant males (Pang et al., 2009; Orvoen et al., 2012), whereas mutant males are more prone to anxiety-like behaviors than mutant females (Hickey et al., 2005; Menalled et al., 2009; Orvoen et al., 2012). In humans, depression is roughly twice as prevalent in women as it is in men, and the reason of this difference is currently unknown (Kornstein et al., 2000).

## HTT, BDNF and mood disorders

The general functions of HTT have been recently reviewed (Zuccato et al., 2010). Among other functions, wild-type HTT regulates the production, transport and release of BDNF, a function that is impaired when HTT is mutated or absent (see below). Microarray studies have shown that *Bdnf*^+/−^ and forebrain-specific *Bdnf*^−/−^ mice have transcription profiles similar to those of patients with HD or R6/2 mice (Strand et al., 2007), suggesting a major role for BDNF in the pathogenesis of HD. BDNF has been implicated in the physiopathology of mood disorders (Autry and Monteggia, 2012), and is thus the most obvious candidate linking HTT to mood disorders.

Wild-type HTT positively regulates *Bdnf* transcription (Cattaneo et al., 2005). Zuccato and colleagues showed that a neuron restrictive silencer element (NRSE) in the *Bdnf* promoter is indirectly targeted by wild-type HTT (Zuccato et al., 2003). Wild-type HTT inhibits the silencing activity of NRSE by sequestering its transcription factor (REST/NRSF) in the cytoplasm, leading to a high rate of *Bdnf* transcription. In contrast, in the context of HD, aberrant accumulation of REST/NRSF is observed in the nucleus, which impairs *Bdnf* transcription. Hence, one of the functions of HTT is to guarantee the sufficient production of BDNF via the sequestration of REST/NRSF. Mutated HTT also interferes with CREB function, which is an activator of *Bdnf* transcription (Zuccato et al., 2010). In agreement with these observations, low *BDNF* expression has been observed in various brain regions of HD patients, including frontal cortex, striatum, hippocampus, substantia nigra, and cerebellum (Zuccato et al., 2001; Seo et al., 2004). In most mouse models of HD, the abundance of BDNF protein (Duan et al., 2003, 2008; Saydoff et al., 2006; Simmons et al., 2009; Xie et al., 2010) and *Bdnf* mRNA is decreased in mutant compared to normal mice in cortex, striatum and hippocampus (Zuccato et al., 2005; Pang et al., 2006; Zajac et al., 2010). It should be noted that striatal neurons express very low amount of BDNF mRNA (Altar et al., 1997). Therefore, the decrease of BDNF mRNA observed in striatum may not be biologically significant (as suggested in Pang et al., 2006). The diminished detection of BDNF at protein level in striatum may be attributed to a transport and secretion defect of neuronal BDNF coming from cortex or substantia nigra (see below). Studies have reported increased BDNF protein levels in anterior cortex or substantia nigra in R6/1 mice: this may be caused by an accumulation of BDNF linked to a transport deficit along corticostriatal or nigrostriatal pathways (Pineda et al., 2005; Pang et al., 2006) (see below).

In addition to controlling the production of *Bdnf* mRNA, wild-type HTT regulates BDNF transport. Gauthier and colleagues found that BDNF transport in microtubules is stimulated by wild-type HTT (Gauthier et al., 2004). In contrast, cortical BDNF transport is impaired both in the presence of low amounts of wild-type HTT and by the expression of mutant HTT (Gauthier et al., 2004). Similar findings have also been demonstrated in hippocampal neurons (Pla et al., 2013). Defects in BDNF vesicular trafficking decrease the activity-dependent release of BDNF from cortical and hippocampal neurons (Gauthier et al., 2004; Pla et al., 2013). This leads to the downregulation of the phosphorylation of AKT and Erk, both of which are regulated downstream of the interaction of BDNF with its receptor, TrkB (Pla et al., 2013).

HTT-mediated transport is microtubule-dependent and involves huntingtin-associated protein-1 (HAP1), which is implicated in axonal transport via its interaction with the microtubule-dependent molecular motors kinesin and dynein, as well as the dynactin subunit p150*^Glued^* (Li et al., 1998; McGuire et al., 2006; Rong et al., 2007). This complex is altered in the absence of HTT, or in the presence of mutated HTT containing an abnormally expanded polyQ tract. This alteration leads to the detachment of BDNF vesicles from microtubules (Gauthier et al., 2004; Zala et al., 2008).

Another argument in favor of the importance of HTT in axonal transport involves HTT phosphorylation. Indeed, HTT function is regulated by phosphorylation and the phosphorylation status of HTT affects BDNF transport and release. Phosphorylation of HTT at serine 421 by AKT and SGK specifically enhances the anterograde transport and release of BDNF at axon terminals (Humbert et al., 2002; Rangone et al., 2004; Colin et al., 2008). HTT is also phosphorylated at serines 1181 and 1201, which are both targets of Cdk5. In contrast to serine 421, dephosphorylation of these sites stimulates transport and release of BDNF (Ben M'Barek et al., 2013).

The density or the function of TrkB, the receptor for BDNF, may also be altered due to defects in transcription and trafficking. Indeed, the transcriptional activity of *TrkB* is low in HD striatal neurons, independent of the production of BDNF (Ginés et al., 2010). Consistent with this finding, low amounts of TrkB are found in the caudate nucleus and/or cortex of HD post-mortem brains (Ginés et al., 2006; Zuccato et al., 2008). In addition to the transport of BDNF-containing vesicles, HTT also transports TrkB-containing vesicles. The retrograde transport of activated TrkB-containing endosomes is low in the presence of mutated HTT, leading to an impairment in neurotrophin signaling in striatal dendrites. This subsequently affects phospho-Erk and c-fos within striatal neurons (Liot et al., 2013). An impairment in Ras/MAPK/ERK1/2 signaling resulting from a low abundance of TrkB may also be associated with the low abundance of p52/p46 Shc docking proteins in striatal cells expressing polyQ-HTT (Ginés et al., 2010). Rab11 is also important for TrkB distribution in dendrites (Lazo et al., 2013) and defects of Rab11 recycling endosomes have been observed in HD (Li et al., 2010).

**Figure 1:**
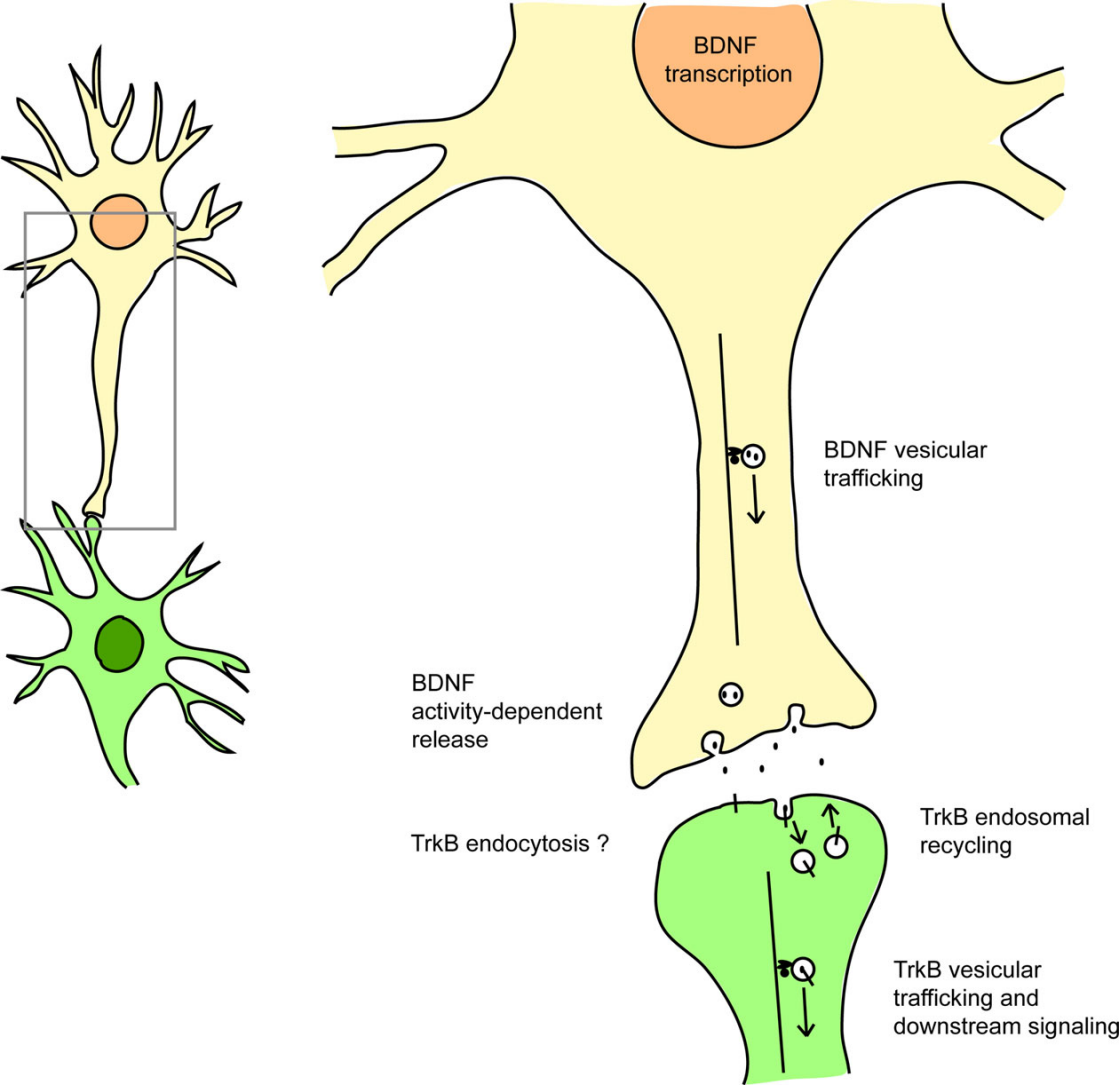
**HTT affects BDNF/TrkB signaling at many levels**. HTT regulates BDNF transcription (via the sequestration of REST/NRSF), BDNF vesicular trafficking (via interaction with the molecular motors dynein and kinesin), the activity-dependent release of BDNF, and also TrkB retrograde vesicular trafficking (via HAP1 interaction). HTT also affects Rab11-dependent endosomal recycling. HTT could also modulate TrkB endocytosis via its interaction with HAP40 and early endosomal trafficking via its interaction with Rab5. See references in the text.

Therefore, by dysregulating transcription, trafficking and signaling at multiple levels, mutant HTT impairs both the ligand and the receptor in the BDNF/TrkB pathway (Figure 1).

However, the hypothesis that the impairment of the BDNF/TrkB pathway alone can lead to mood disorder is still a matter of debate. Most studies support the conclusion that an impairment in the production of BDNF is not associated with profound changes to depression-related behavior (Autry and Monteggia, 2012). Yet, in the forced swim test and in the sucrose preference test, female mice lacking BDNF show a high degree of depressive-like behavior (Monteggia et al., 2007). This is similar to the situation in HD, in which females are more prone to depressive-like behaviors than males. In R6/1 mice, the production of BDNF is mostly affected in females, because the abundance of a large number of *Bdnf* isoforms (BDNF I, II, III, IV and VI) is low in females, whereas in males only BDNF I and VI transcripts are affected (Zajac et al., 2010).

In contrast, there is a clear link between anxiety-related behavior and BDNF signaling. KI mice that express BDNF containing a point mutation (valine 66 to methionine substitution, Val/Met 66) that mimics a mutation found in human populations, show impairment in BDNF secretion and exhibit high levels of anxiety-like behavior in open field and elevated plus maze tests (Chen et al., 2006). Genetic or pharmacological inhibition of TrkB also results in the modification of anxiety-related behavior (Bergami et al., 2008; Cazorla et al., 2010). Conversely, antidepressants such as SSRIs, tricyclic molecules, or monoamine oxidase-A inhibitors are able to rapidly activate BDNF/TrkB signaling (Saarelainen et al., 2003; Rantamäki et al., 2007).

New discoveries have highlighted a role for BDNF production and HTT in cell types other than neurons. BDNF produced by astrocytes can modify mood-related behavior in mice (Quesseveur et al., 2013). Expression of a mutated fragment of HTT in astrocytes causes age-dependent neurological symptoms, including motor function deficits (Bradford et al., 2009). Anxiety or depressive-related behaviors were not examined in these mice and such a study would be of interest.

Finally, many studies demonstrate that the efficacy of antidepressant treatments depends on fully functioning BDNF production and signaling (Monteggia et al., 2004; Malberg and Blendy, 2005; Monteggia et al., 2007; Hu and Russek, 2008; Li et al., 2008). Thus, restoration of the “normal” levels of BDNF could be a therapy for HD patients. Normal levels of BDNF can be restored by physical exercise in HD models. According to Zajac et al. (2010), the abundance of total *Bdnf* mRNA that is low in the hippocampus of both male and female R6/1 mice is significantly increased by wheel-running activity in female R6/1 mice and wild-type mice (but not in male R6/1 mice). Environmental enrichment is another method that promotes BDNF production. Environmental enrichment is associated with a high abundance of *Bdnf* mRNA in the hippocampus of male wild-type animals (Zajac et al., 2010) and environmental stimulation benefits patients with HD (Sullivan et al., 2001).

## HTT and hippocampal adult neurogenesis

The amount of neurogenesis in the hippocampal dentate gyrus (DG) is associated with anxiety state. Intriguingly, both high or low levels of DG neurogenesis have been associated with anxiogenic behavior, suggesting that an optimal level of DG neurogenesis is important to maintain a normal anxiety-related state (Fuss et al., 2010). Low levels of neurogenesis alone are not sufficient to trigger a depression-like phenotype; however, neurogenesis is necessary for some of the beneficial effects of anti-depressant treatments (Samuels and Hen, 2011; Mendez-David et al., 2014).

Neurogenesis in the subventricular zone (SVZ) is increased in post-mortem HD brain tissue (Curtis et al., 2003). However, neurogenesis in the DG has not been examined in HD patients. In HD rodent models, adult neurogenesis is altered in the hippocampus whereas no significant alteration of neurogenesis in the SVZ has been observed (Gil et al., 2005; Phillips et al., 2005; Lazic et al., 2006;). This suggests that the mechanism with which HTT controls neurogenesis is region-specific. The discrepancy between patients and mice regarding neurogenesis in the SVZ could be explained by the fact that the loss of striatal neurons is more detrimental for humans than for mice; thus, a compensatory activation of neurogenesis in the SVZ could be triggered in humans but not in mice (Curtis et al., 2003).

HD mouse models show defects in cellular proliferation in the DG, however these defects occur during the late stages of disease progression. In the DG of R6/1 mice, cell proliferation is low only when mice already exhibit motor deficits (Lazic et al., 2004, 2006; Walker et al., 2011). Similarly, YAC 128 mice show normal cell proliferation in the DG prior to the onset of motor deficits. However, these mice have 26% fewer proliferating cells in the DG when they are 18 months old compared to wild-type mice (Simpson et al., 2011). No defect in cellular proliferation is observed for *Hdh^Q111/Q111^* mice before the onset of motor deficits, and similarly, cellular proliferation in the loss of function *CaMKCreER^*T*2^*; *Htt^flox/flox^* mice is not affected (Orvoen et al., 2012; Pla et al., 2013).

In contrast to proliferation, the differentiation and survival of new-born DG neurons are affected during the early stages of disease progression, before the onset of motor deficits. YAC128 mice show morphological alterations to DG immature neurons (Simpson et al., 2011). Male but not female *Hdh^Q111/Q111^* mice have defects of dendritic arborization of new-born DG neurons (Orvoen et al., 2012). This correlates well with anxiety-related behavior because only male *Hdh^Q111/Q111^* mice show anxiety-like behavior (Orvoen et al., 2012). This correlation extends to the loss-of-function *CaMKCreER^*T*2^; Htt^flox/flox^*model, in which anxiety-like behavior is also associated with defects of dendritic arborization and the poor long-term survival of new-born DG neurons (Pla et al., 2013). Conversely, in KI mice that express HTT containing non-phosphorylatable serines 1181 and 1201, an anxiolytic-like behavior is associated with an increase of dendritic arborization and survival of newborn DG neurons (Ben M'Barek et al., 2013).

The deletion of *Htt* in mature cortical and hippocampal neurons affects only anxiety-related behavior and not depressive-related behavior (Pla et al., 2013). Female *Hdh^Q111/Q111^* mice have a depressive-like phenotype without perturbation of neurogenesis (Orvoen et al., 2012). Hence in HD, depression could be related to defects in other neuronal systems, such as raphe serotonergic neurons (see below).

The origin of defects of hippocampal neurogenesis may be non-cell autonomous, as is the case for *CaMKCreER^*T*2^*; *Htt^flox/flox^* mice. In these mice, HTT is specifically depleted in adult mature cortical and hippocampal neurons. However, new-born neurons show defects in dendritogenesis and survival, despite the fact that *Htt* is still expressed in these neurons (Pla et al., 2013). The phenotype of these new-born neurons is similar to that of TrkB-depleted new-born neurons (Bergami et al., 2008). This suggests that BDNF may be involved in the non-cell autonomous effect of mature neurons on new-born neurons.

Natural or induced changes to the abundance of BDNF have been associated with changes to adult hippocampal neurogenesis in several studies (Lee et al., 2002; Duman, 2004; Sairanen et al., 2005). However, although physical exercise promotes the production of BDNF, this is not associated with an improvement to hippocampal neurogenesis in various HD models (Kohl et al., 2007; Renoir et al., 2012). Thus, in the context of HD, the production of BDNF does not seem to restore normal hippocampal neurogenesis. The exercise-dependent stimulation of neurogenesis depends on PI3K-Akt signaling (Bruel-Jungerman et al., 2009), which acts downstream of BDNF/TrkB. Hence, the absence of a pro-neurogenic response to exercise in these HD models could be linked to the aforementioned defects of BDNF/TrkB signaling. Other players may also be involved, such as the serotonergic system.

## HTT and the serotonergic signaling

Dysregulation of the raphe serotonergic system has been implicated as an important factor in mood disorders, including depression (Donaldson et al., 2013). Similarly, several observations point to a dysregulation of this system in HD. A study using transcranial ultrasound found a correlation between raphe echogenicity and the level of depression in patients with HD (Krogias et al., 2011). Concentrations of 5-hydroxytryptamine (5-HT) and its metabolite 5-hydroxyindolacetic acid (5-HIAA), which are often low in patients with major depression, are also low in HD patients (Caraceni et al., 1977; Jongen et al., 1980), whereas the activity of monoamine oxydase A enzyme activity (which catalyzes the breakdown of 5-HT) is high (Richards et al., 2011). Furthermore, 5-HT binding to its receptor is also impaired, as assessed in post-mortem HD brains (Waeber and Palacios, 1989; Steward et al., 1993; Wong et al., 1996). The Htr1A and Htr1B receptor subtypes appear to be particularly affected (Castro et al., 1998; Yohrling et al., 2002). The expression of serotonergic receptors is perturbed in murine HD models (Pang et al., 2009; Renoir et al., 2012). The expression of *Htr1a*, *Htr1b*, and *Htr2a* is low, whereas the expression of *Htr2c* is normal. Lower concentrations of serotonin (and also of 5-HIAA), dopamine, and noradrenaline were found in the striatum, cortex, and hippocampus of R6/1 mice compared to wild-type mice (Renoir et al., 2011).

The behavior of R6/1 mice, including its sex-specific component, resembles that of *Htr1a*^−/−^, or *Htr1b*^−/−^, or *SERT*^−/−^ mice (Gross et al., 2000; Mayorga et al., 2001; Holmes et al., 2002; Lira et al., 2003; Jones and Lucki, 2005). Gene expression studies have found changes to the expression of *Htr1a*, *Htrb1*, and *SERT* in the brains of R6/1 mice (Pang et al., 2009), suggesting that alterations of mood behavior in HD mice could be caused, at least in part, by a low abundance of these 5-HT receptors and this 5-HT transporter. The expression of *Htr1b* is lower in R6/1 females than in males (Pang et al., 2009), making *Htr1b* an obvious candidate to explain sex-specific differences in mood behavior. Female R6/1 mice are more sensitive than males to the Htr1a receptor agonist 8-OH DPAT in the 8-OH DPAT-induced hypothermia test, suggesting sex-specific Htr1a hypersensitivity (Renoir et al., 2011, 2012). SSRI sertraline treatment in female R6/1 mice was associated with both a reduction in depressive-like behavior and in the hypersensitivity of Htr1a autoreceptors (Renoir et al., 2012). This is consistent with observations in humans because sertraline is more effective for the treatment of chronic depression in women than in men (Kornstein et al., 2000).

It is not known if mutant HTT directly affects the transcription of genes encoding serotonergic receptors or transporters. However, serotonergic signaling may affect the expression of *HTT*. Indeed, in *Htr1a/Htr1b* double KO mice, which have a hyperserotonergic phenotype and show anxiety-related behavior, the expression of *Htt* and other HD-related genes is perturbed (Xia et al., 2012).

Serotonergic signaling can affect hippocampal neurogenesis. This effect depends on the serotonin receptor involved, although the overall effect is pro-neurogenic (Klempin et al., 2010). Therefore, the low abundance of various serotonin receptors that is observed in the hippocampus in HD models (see above) could at least partially explain the defects in DG neurogenesis. TrkB is expressed on serotonergic raphe neurons and BDNF produced in the hippocampus can be retrogradely transported to the raphe (Anderson et al., 1995; Madhav et al., 2001). BDNF/TrkB signaling affects the function of serotonergic neurons. For example, *Bdnf*^+/−^ mice show changes in serotonergic innervations of the cortex, hypothalamus, and hippocampus (Lyons et al., 1999; Luellen et al., 2007) and it would be interesting to examine the densities of serotonergic innervations in HD models.

## HTT and the HPA axis

The hypothalamic-pituitary-adrenal axis (HPA) is the main regulator of the stress response. In the HPA axis, cortisol secretion is controlled by the hypothalamic corticotrophin releasing hormone (CRH) and pituitary adrenocorticotropic hormone (ACTH). Stress leads to the hyper-secretion of corticosteroids by the adrenal glands and this is associated with a high risk of anxiety and depression. Therefore, changes to the activity of the HPA axis are common in patients with depression (Antonijevic, 2006). Chronic treatment of rodents with corticosterone induces anxiety- and depression-related behavior (Ardayfio and Kim, 2006; Gourley et al., 2008; Murray et al., 2008; David et al., 2009). The acute or chronic administration of corticosterone in mice is associated with impairment of neurogenesis and poor neuronal survival in the hippocampus (Cameron and Gould, 1994; Karishma and Herbert, 2002; Murray et al., 2008; David et al., 2009).

HPA hyperactivity has been observed in HD patients (Heuser et al., 1991; Leblhuber et al., 1995; Björkqvist et al., 2006; Aziz et al., 2009). In saliva samples taken early in the morning, pre-symptomatic HD carriers show higher cortisol concentrations than controls (Van Duijn et al., 2010). During the early stages of HD, salivary concentrations of cortisol in the morning are higher in depressed patients than in non-depressed patients (Shirbin et al., 2013). High concentrations of cortisol have been measured in transgenic R6/2 mice (Björkqvist et al., 2006) but also specifically in female R6/1 mice subjected to physiological or pharmacological stresses (Du et al., 2012). This may explain the tendency of HD female mice to have more depressive-like phenotypes than HD male mice in various models. Furthermore, testosterone has a protective effect against HPA hyperactivity (Rubinow et al., 2005).

## Conclusions and perspectives

**Figure 2:**
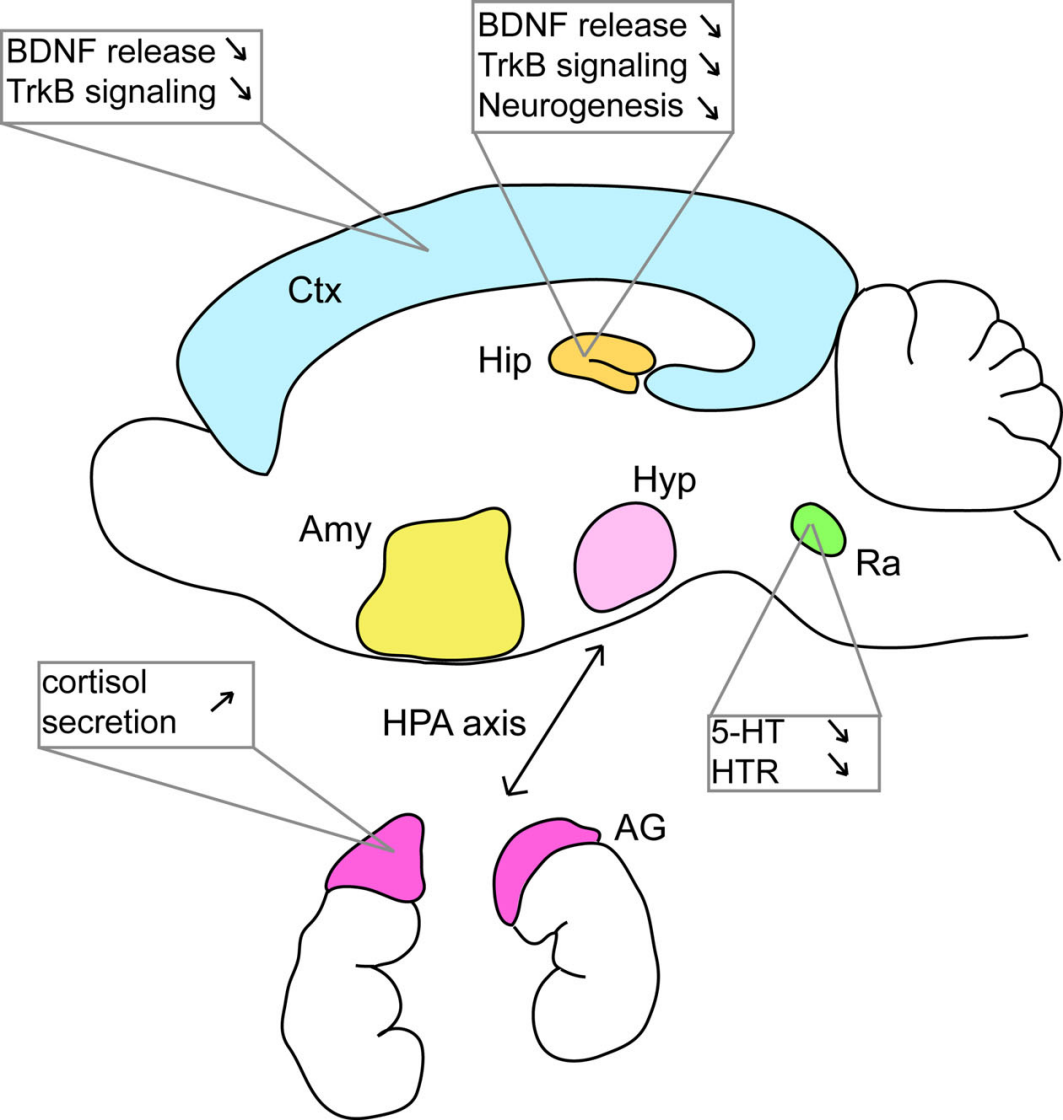
**Many defects at various locations in the brain of HD murine models can be linked to mood disorders**. Studies in mouse models of HD have shown that BDNF/TrkB signaling is altered in various brain regions. Alterations of the serotonergic system and of the HPA axis have also been documented. AG, adrenal gland; Amy, amygdala; Ctx, cortex; Hip, hippocampus; Hyp, hypothalamus; Ra, raphe nuclei. See references in the text.

Studies of anxiety and depression have mainly focused on the hippocampus, serotonergic signaling, and to a lesser extent, the HPA axis (Figure 2). However, depression involves perturbations of many other regions such as the prefrontal cortex, the cingulate cortex, the striatum, the amygdala, and the thalamus (Nestler et al., 2002). In the context of HD, research has historically concentrated on perturbations to the striatum with a focus on motor control. However, striatal alterations have also been implicated in HD early cognitive defects like executive dysfunction (Peinemann et al., 2005) and future studies may also implicate striatal dysfunction in mood disorders in HD patients. All these regions are highly interconnected and mood disorders alter these circuits. It seems reasonable to think that depression and anxiety that are associated with HD have several origins within these circuits. Targeted deletion or KI mouse models could be used to answer this question. Recently, Du et al. (2013) proposed that dysfunction of the HPA axis could trigger a cascade of events participating to mood disorders in patients with HD.

Defects in adult hippocampal neurogenesis have also been implicated in cognitive alteration of learning and memory, which are also present in HD. Therefore, the study of hippocampal neurogenesis will benefit not only our understanding of mood alterations in patients with HD, but also our understanding of cognitive deficits in these patients. In addition, BDNF has been implicated in hippocampal neurogenesis but is also involved in the development and maintenance of dendritic spines (Tyler and Pozzo-Miller, 2003; Alonso et al., 2004; Von Bohlen und Halbach et al., 2006, 2008), as well as synaptic plasticity (Gomez-Palacio-Schjetnan and Escobar, 2013). Thus, defects of BDNF/TrkB signaling that are described for HD models may also affect cognition. In this regard, it is interesting to note that depression is also associated with poor cognitive performance in patients with HD (Smith et al., 2012).

HTT controls the intracellular trafficking of other ligands and receptors besides BDNF and TrkB. Perturbation of this trafficking in the presence of mutated HTT may also contribute to the pathology of mood disorders For example, polyQ-HTT alters GABA_A_R vesicle trafficking, resulting in the loss of GABA_A_R surface delivery and in a low inhibitory synaptic response (Twelvetrees et al., 2010; Yuen et al., 2012). GABA_A_R dysfunction is implicated in mood disorders (Rudolph and Möhler, 2014). GABA interneurons are inhibited by 5-HT1A receptors in basolateral amygdala, an area implicated in anxiety and fear processing (Rainnie, 1999). Moreover, NMDA receptor signaling pathways are perturbed in HD patients (Young et al., 1988). In transgenic YAC models of HD, alteration of NMDA receptor activity that affects long-term potentiation (LTP) is an early event prior to any motor or cognitive defects (Hodgson et al., 1999; Milnerwood and Raymond, 2007).

Another level of complexity that remains to be explored is the role of the various fragments of HTT that are generated during the progression of HD. Mutated HTT is cleaved by caspases. In particular, caspase-6 cleavage represents an important event mediating neuronal dysfunction and neurodegeneration (Gafni et al., 2004; Graham et al., 2006). YAC mice expressing a caspase-6-resistant mutant HTT exhibit only a moderate impairment of motor functions but also a modest depressive-like phenotype (Pouladi et al., 2009). These data suggest that caspase-6-mediated cleavage of HTT is an important trigger of depressive behavior. As some N-terminal fragments do not recapitulate full-length HTT functions, proteolysis event could be linked to the inactivation of HTT protein function.

BDNF release and signaling is decreased in the hippocampus in the context of HD, which is probably associated with the loss of HTT function in vesicular transport. However, BDNF signaling is increased in the amygdala and in the nucleus accumbens during major depression or stress disorders (Berton et al., 2006; Krishnan et al., 2007; Yu and Chen, 2011). Whether this is also true for depressed or stressed patients with HD and HD mouse models remains to be explored. If the activity of BDNF signaling is high in the amygdala and in the nucleus accumbens in the context of HD, then this would suggest that mutated HTT has region-specific effects on BDNF signaling. Little is known about the functions of HTT in the “fear” and in the reward systems where amygdala and nucleus accumbens are implicated respectively.

The underlying unresolved question is as follows: are the depressive symptoms observed for patients with HD different from those of individuals without HD? In other words, are the mechanisms at the basis of depression in patients with HD specific to this disease? The loss of HTT specifically in mature hippocampal neurons affects anxio-depressive behavior and the phosphorylation of HTT at S1181/1201 also appears to regulate mood-related behavior in mouse models of HD. These observations suggest that HTT is part of the physiological cascade controlling anxio-depressive behavior. Hence, the interest of studying HTT extends beyond HD. HTT could constitute a novel target for therapy in anxiety and depression both in patients with HD and in the general population. This could be important given that the effectiveness of currently available drugs is highly variable for many patients (Samuels and Hen, 2011; Kupfer et al., 2012).

### Conflict of interest statement

The authors declare that the research was conducted in the absence of any commercial or financial relationships that could be construed as a potential conflict of interest.

## References

[B1] Almqvist E. W. Brinkman R. R. Wiggins S. Hayden M. R. The Canadian Collaborative Study of Predictive Testing. (2003). Psychological consequences and predictors of adverse events in the first 5 years after predictive testing of HD. Clin. Genet. 64, 300–309 10.1034/j.1399-0004.2003.00157.x 12974735

[B2] Alonso M. Medina J. H. Pozzo-Miller L. (2004). ERK1/2 activation is necessary for BDNF to increase dendritic spine density in hippocampal CA1 pyramidal neurons. Learn. Mem. 11, 172–178 10.1101/lm.67804 15054132 PMC379687

[B3] Altar C. A. Cai N. Bliven T. Juhasz M. Conner J. M. Acheson A. L. (1997). Anterograde transport of brain-derived neurotrophic factor and its role in the brain. Nature 389, 856–860 10.1038/39885 9349818

[B4] Anderson K. D. Alderson R. F. Altar C. A. DiStefano P. S. Corcoran T. L. Lindsay R. M. Wiegand S. J. (1995). Differential distribution of exogenous BDNF, NGF and NT-3 in the brain corresponds to the relative abundance and distribution of high-affinity neurotrophin receptors. J. Comp. Neurol. 357, 296–317 7665731 10.1002/cne.903570209

[B5] Antonijevic I. A. (2006). Depressive disorders: is it time to endorse different pathophysiologies? Psychoneuroendocrinology 31, 1–15 10.1016/j.psyneuen.2005.04.004 15950391

[B6] Ardayfio P. Kim K.-S. (2006). Anxiogenic-like effect of chronic corticosterone in the light-dark emergence task in mice. Behav. Neurosci. 120, 249–256 10.1037/0735-7044.120.2.249 16719689

[B7] Autry A. E. Monteggia L. M. (2012). Brain-derived neurotrophic factor and neuropsychiatric disorders. Pharmacol. Rev. 64, 238–258 10.1124/pr.111.005108 22407616 PMC3310485

[B182] Aziz N. A. Pijl H. Frölich M. van der Graaf A. W. Roelfsema F. Roos R. A. (2009). Increased hypothalamic-pituitary-adrenal axis activity in Huntington's disease. J. Clin. Endocrinol. Metab. 94, 1223–1228 10.1210/jc.2008-2543 19174491

[B8] Ben M'Barek K. Pla P. Orvoen S. Benstaali C. Godin J. D. Gardier A. M. (2013). Huntingtin mediates anxiety/depression-related behaviors and hippocampal neurogenesis. J. Neurosci. 33, 8608–8620 10.1523/J.NEUROSCI.5110-12.2013 23678106 PMC6618836

[B9] Bergami M. Rimondini R. Santi S. Blum R. Götz M. Canossa M. (2008). Deletion of TrkB in adult progenitors alters newborn neuron integration into hippocampal circuits and increases anxiety-like behavior. Proc. Natl. Acad. Sci. U.S.A. 105, 15570–15575 10.1073/pnas.0803702105 18832146 PMC2557028

[B10] Berrios G. E. Wagle A. C. Markova I. S. Wagle S. A. Ho L. W. Rubinsztein D. C. (2001). Psychiatric symptoms and CAG repeats in neurologically asymptomatic Huntington's disease gene carriers. Psychiatry Res. 102, 217–225 10.1016/S0165-1781(01)00257-8 11440772

[B11] Berton O. McClung C. A. Dileone R. J. Krishnan V. Renthal W. Russo S. J. (2006). Essential role of BDNF in the mesolimbic dopamine pathway in social defeat stress. Science 311, 864–868 10.1126/science.1120972 16469931

[B12] Björkqvist M. Petersén A. Bacos K. Isaacs J. Norlén P. Gil J. (2006). Progressive alterations in the hypothalamic-pituitary-adrenal axis in the R6/2 transgenic mouse model of Huntington's disease. Hum. Mol. Genet. 15, 1713–1721 10.1093/hmg/ddl094 16613897

[B13] Bradford J. Shin J.-Y. Roberts M. Wang C.-E. Li X.-J. Li S. (2009). Expression of mutant huntingtin in mouse brain astrocytes causes age-dependent neurological symptoms. Proc. Natl. Acad. Sci. U.S.A. 106, 22480–22485 10.1073/pnas.0911503106 20018729 PMC2799722

[B14] Bruel-Jungerman E. Veyrac A. Dufour F. Horwood J. Laroche S. Davis S. (2009). Inhibition of PI3K-Akt signaling blocks exercise-mediated enhancement of adult neurogenesis and synaptic plasticity in the dentate gyrus. PLoS ONE 4:e7901 10.1371/journal.pone.0007901 19936256 PMC2775944

[B15] Cameron H. A. Gould E. (1994). Adult neurogenesis is regulated by adrenal steroids in the dentate gyrus. Neurosci. 61, 203–209 7969902 10.1016/0306-4522(94)90224-0

[B16] Caraceni T. Calderini G. Consolazione A. Riva E. Algeri S. Girotti F. (1977). Biochemical aspects of Huntington's chorea. J. Neurol. Neurosurg. Psychiatry 40, 581–587 10.1136/jnnp.40.6.581 143508 PMC492765

[B17] Castro M. E. Pascual J. Romon T. Pazos A. (1998). 5-HT 1B receptor binding in degenerative movement disorders. Brain Res. 790, 323–328 9593971 10.1016/s0006-8993(97)01566-7

[B18] Cattaneo E. Zuccato C. Tartari M. (2005). Normal huntingtin function: an alternative approach to Huntington's disease. Nat. Rev. Neurosci. 6, 919–930 10.1038/nrn1806 16288298

[B19] Cazorla M. Jouvenceau A. Rose C. Guilloux J.-P. Pilon C. Dranovsky A. (2010). Cyclotraxin-B, the first highly potent and selective TrkB inhibitor, has anxiolytic properties in mice. PLoS ONE 5:e9777 10.1371/journal.pone.0009777 20333308 PMC2841647

[B20] Chari A. Quiraishi S. H. Jainer A. K. (2003). Fluoxetine-induced exacerbation of chorea in Huntington's disease ? A case report. Pharmacopsychiatry 36, 41–43 10.1055/s-2003-38093 12649776

[B21] Chen Z. Y. Jing D. Bath K. G. Ieraci A. Khan T. Siao C. J. (2006). Genetic variant BDNF (Val66Met) polymorphism alters anxiety-related behavior. Science 314, 140–143 10.1126/science.1129663 17023662 PMC1880880

[B22] Chiu C.-T. Liu G. Leeds P. Chuang D.-M. (2011). Combined treatment with the mood stabilizers lithium and valproate produces multiple beneficial effects in transgenic mouse models of Huntington's disease. Neuropsychopharmacology 36, 2406–2421 10.1038/npp.2011.128 21796107 PMC3194069

[B23] Ciamei A. Morton A. J. (2008). Rigidity in social and emotional memory in the R6/2 mouse model of Huntington's disease. Neurobiol. Learn. Mem. 89, 533–544 10.1016/j.nlm.2007.10.009 18069020

[B24] Colin E. Zala D. Liot G. Rangone H. Borrell-Pagès M. Li X.-J. (2008). Huntingtin phosphorylation acts as a molecular switch for anterograde/retrograde transport in neurons. EMBO J. 27, 2124–2134 10.1038/emboj.2008.133 18615096 PMC2516882

[B25] Craufurd D. Thompson J. C. Snowden J. S. (2001). Behavioral change in Huntington disease. Neuropsychiatry Neuropsychol. Behav. Neurol. 14, 219–226 11725215

[B26] Curtis M. A. Penney E. B. Pearson A. G. van Roon-Mom W. M. C. Butterworth N. J. Dragunow M. (2003). Increased cell proliferation and neurogenesis in the adult human Huntington's disease brain. Proc. Natl. Acad. Sci. U.S.A. 100, 9023–9027 10.1073/pnas.1532244100 12853570 PMC166431

[B27] David D. J. Samuels B. A. Rainer Q. Wang J. Marsteller D. Mendez I. (2009). Neurogenesis-dependent and -independent effects of fluoxetine in an animal model of anxiety/depression. Neuron 62, 479–493 10.1016/j.neuron.2009.04.017 19477151 PMC2759281

[B28] Detke M. J. Rickels M. Lucki I. (1995). Active behaviors in the rat forced swimming test differentially produced by serotonergic and noradrenergic antidepressants. Psychopharmacology 121, 66–72 8539342 10.1007/BF02245592

[B29] Dietrich P. Shanmugasundaram R. Shuyu E. Dragatsis I. (2009). Congenital hydrocephalus associated with abnormal subcommissural organ in mice lacking huntingtin in Wnt1 cell lineages. Hum. Mol. Genet. 18, 142–150 10.1093/hmg/ddn324 18838463 PMC3298867

[B30] Donaldson Z. R. Nautiyal K. M. Ahmari S. E. Hen R. (2013). Genetic approaches for understanding the role of serotonin receptors in mood and behavior. Curr. Opin. Neurobiol. 23, 399–406 10.1016/j.conb.2013.01.011 23385115 PMC3652904

[B31] Dragatsis I. Levine M. S. Zeitlin S. (2000). Inactivation of Hdh in the brain and testis results in progressive neurodegeneration and sterility in mice. Nat. Genet. 26, 300–306 10.1038/81593 11062468

[B32] Du X. Leang L. Mustafa T. Renoir T. Pang T. Y. Hannan A. J. (2012). Environemental enrichement rescues female-specific hyperactivity of the hypothalamic-pituitary-adrenal axis in a model of Huntington's disease. Transl. Psychiatry 2:e133 10.1038/tp.2012.58 22760557 PMC3410631

[B33] Du X. Pang T. Y. Hannan A. J. (2013). A tale of two maladies ? Pathogenesis of depression with and without the Huntington's Disease Gene Mutation. Front. Neurol. 4:81 10.3389/fneur.2013.00081 23847583 PMC3705171

[B34] Duan W. Guo Z. Jiang H. Ware M. Li X.-J. Mattson M. P. (2003). Dietary restriction normalizes glucose metabolism and BDNF levels, slows disease progression, and increases survival in huntingtin mutant mice. Proc. Natl. Acad. Sci. U.S.A. 100, 2911–2916 10.1073/pnas.0536856100 12589027 PMC151440

[B35] Duan W. Peng Q. Masuda N. Ford E. Tryggestad E. Ladenheim B. (2008). Sertraline slows disease progression and increases neurogenesis in N171-82Q mouse model of Huntington's disease. Neurobiol. Dis. 30, 312–322 10.1016/j.nbd.2008.01.015 18403212 PMC3683653

[B36] Duff K. Paulsen J. S. Beglinger L. J. Langbehn D. R. Stout J. C. (2007). Psychiatric symptoms in Huntington's disease before diagnosis?: the predict-HD study. Biol. Psychiatry 62, 1341–1346 10.1016/j.biopsych.2006.11.034 17481592

[B38] Duman R. S. (2004). Role of neurotrophic factors in the etiology and treatment of mood disorders. Neuromolecular Med. 5, 11–25 10.1385/NMM:5:1:011 15001809

[B39] Duyao M. P. Auerbach A. B. Ryan A. Persichetti F. Barnes G. T. McNeil S. M. (1995). Inactivation of the Mouse Huntington's disease gene homolog Hdh. Science 269, 6182–6195 7618107 10.1126/science.7618107

[B40] Epping E. A. Mills J. A. Beglinger L. J. Fiedorowicz J. G. Craufurd D. Smith M. M. (2013). Characterization of depression in prodromal Huntington disease in the neurobiological predictors of HD (PREDICT-HD) study. J. Psychiatr. Res. 47, 1423–1431 10.1016/j.jpsychires.2013.05.026 23790259 PMC3808084

[B41] Epping E. A. Paulsen J. S. (2011). Depression in the early stages of Huntington disease. Neurodegener. Dis. Manag. 1, 407–414 10.2217/nmt.11.45 22942903 PMC3430381

[B42] File S. E. Mahal A. Mangiarini L. Bates G. P. (1998). Striking changes in anxiety in Huntington's disease transgenic mice. Brain Res. 805, 234–240 10.1016/S0006-8993(98)00736-7 9733972

[B43] Folstein S. E. Chase G. A. Wahl W. E. McDonnell A. M. Folstein M. F. (1987). HD in Maryland?: clinical aspects of racial variation. Am. J. Hum. Genet. 41, 168–179 2956881 PMC1684204

[B44] Frank S. (2010). Tetrabenazine: the first approved drug for the treatment of chorea in US patients with Huntington's disease. Neuropsychiatr. Dis. Treat. 6, 657–665 10.2147/NDT.S6430 20957126 PMC2951749

[B45] Fuss J. Ben Abdallah N. M. B. Hensley F. W. Weber K.-J. Hellweg R. Gass P. (2010). Deletion of running-induced hippocampal neurogenesis by irradiation prevents development of an anxious phenotype in mice. PLoS ONE 5:e12769 10.1371/journal.pone.0012769 20862278 PMC2940841

[B46] Gafni J. Hermel E. Young J. E. Wellington C. L. Hayden M. R. Ellerby L. M. (2004). Inhibition of calpain cleavage of huntingtin reduces toxicity: accumulation of calpain/caspase fragments in the nucleus. J. Biol. Chem. 279, 20211–20220 10.1074/jbc.M401267200 14981075

[B47] Gauthier L. R. Charrin C. Dompierre J. P. Borrell-Pagès M. Cordelières F. P. De Mey J. (2004). Huntingtin controls neurotrophic support and survival of neurons by enhancing BDNF vesicular transport along microtubules. Cell 118, 127–138 10.1016/j.cell.2004.06.018 15242649

[B48] Gil J. M. A. C. Mohapel P. Araújo I. M. Popovic N. Li J.-Y. Brundin P. (2005). Reduced hippocampal neurogenesis in R6/2 transgenic Huntington's disease mice. Neurobiol. Dis. 20, 744–751 10.1016/j.nbd.2005.05.006 15951191

[B49] Ginés S. Bosch M. Marco S. Gavaldà N. Díaz-Hernández M. Lucas J. J. (2006). Reduced expression of the TrkB receptor in Huntington's disease mouse models and in human brain. Eur. J. Neurosci. 23, 649–658 10.1111/j.1460-9568.2006.04590.x 16487146

[B50] Ginés S. Paoletti P. Alberch J. (2010). Impaired TrkB-mediated ERK1/2 activation in huntington disease knock-in striatal cells involves reduced p52/p46 Shc expression. J. Biol. Chem. 285, 21537–21548 10.1074/jbc.M109.084202 20442398 PMC2898383

[B52] Gomez-Palacio-Schjetnan A. Escobar M. L. (2013). Neurotrophins and synaptic plasticity. Curr. Top. Behav. Neurosci. 15, 117–136 10.1007/7854_2012_231 23519767

[B53] Gourley S. L. Kiraly D. D. Howell J. L. Olausson P. Taylor J. R. (2008). Acute hippocampal brain-derived neurotrophic factor restores motivational and forced swim performance after corticosterone. Biol. Psychiatry 64, 884–890 10.1016/j.biopsych.2008.06.016 18675955 PMC2633780

[B54] Graham R. K. Deng Y. Slow E. J. Haigh B. Bissada N. Lu G. (2006). Cleavage at the caspase-6 site is required for neuronal dysfunction and degeneration due to mutant huntingtin. Cell 125, 1179–1191 10.1016/j.cell.2006.04.026 16777606

[B55] Gray M. Shirasaki D. I. Cepeda C. André V. M. Wilburn B. Lu X.-H. (2008). Full-length human mutant huntingtin with a stable polyglutamine repeat can elicit progressive and selective neuropathogenesis in BACHD mice. J. Neurosci. 28, 6182–6195 10.1523/JNEUROSCI.0857-08.2008 18550760 PMC2630800

[B56] Gross C. Santarelli L. Brunner D. Zhuang X. Hen R. (2000). Altered fear circuits in 5-HT(1A) receptor KO mice. Biol. Psychiatry 48, 1157–1163 10.1016/S0006-3223(00)01041-6 11137057

[B57] Grote H. E. Bull N. D. Howard M. L. van Dellen A. Blakemore C. Bartlett P. F. (2005). Cognitive disorders and neurogenesis deficits in Huntington's disease mice are rescued by fluoxetine. Eur. J. Neurosci. 22, 2081–2088 10.1111/j.1460-9568.2005.04365.x 16262645

[B58] Hackam S. Singaraja R. Wellington C. L. Metzler M. McCutcheon K. Zhang T. (1998). The influence of huntingtin protein size on nuclear localization and cellular toxicity. J. Cell Biol. 141, 1097–1105 9606203 10.1083/jcb.141.5.1097 PMC2137174

[B59] Heng M. Y. Detloff P. J. Albin R. L. (2008). Rodent genetic models of Huntington disease. Neurobiol. Dis. 32, 1–9 10.1016/j.nbd.2008.06.005 18638556

[B60] Heuser I. J. Chase T. N. Mouradian M. M. (1991). The limbic-hypothalamic-pituitary-adrenal axis in Huntington's disease. Biol. Psychiatry 30, 943–952 1660734 10.1016/0006-3223(91)90007-9

[B61] Hickey M. A. Gallant K. Gross G. G. Levine M. S. Chesselet M.-F. (2005). Early behavioral deficits in R6/2 mice suitable for use in preclinical drug testing. Neurobiol. Dis. 20, 1–11 10.1016/j.nbd.2005.01.024 16137562

[B62] Hickey M. A. Kosmalska A. Enayati J. Cohen R. Zeitlin S. Levine M. S. (2008). Extensive early motor and non-motor behavioral deficits are followed by striatal neuronal loss in knock-in Huntington's disease mice. Neuroscience 157, 280–295 10.1016/j.neuroscience.2008.08.041 18805465 PMC2665298

[B63] Hodgson J. G. Agopyan N. Gutekunst C.-A. Leavitt B. R. LePiane F. Singaraja R. (1999). A YAC mouse model for huntington's disease with full-length mutant huntingtin, cytoplasmic toxicity, and selective striatal neurodegeneration. Neuron 23, 181–192 10402204 10.1016/s0896-6273(00)80764-3

[B64] Holmes A. Yang R. J. Murphy D. L. Crawley J. N. (2002). Evaluation of antidepressant-related behavioral responses in mice lacking the serotonin transporter. Neuropsychopharmacology 27, 914–923 10.1016/S0893-133X(02)00374-3 12464448

[B65] Hu Y. Russek S. J. (2008). BDNF and the diseased nervous system: a delicate balance between adaptive and pathological processes of gene regulation. J. Neurochem. 105, 1–17 10.1111/j.1471-4159.2008.05237.x 18208542

[B183] Humbert S. Bryson E. A. Cordelières F. P. Connors N. C. Datta S. R. Finkbeiner S. (2002). The IGF-1/Akt pathway is neuroprotective in Huntington's disease and involves Huntingtin phosphorylation by Akt. Dev. Cell. 2, 831–837 10.1016/S1534-5807(02)00188-0 12062094

[B66] Jones M. D. Lucki I. (2005). Sex differences in the regulation of serotonergic transmission and behavior in 5-HT receptor knockout mice. Neuropsychopharmacology 30, 1039–1047 10.1038/sj.npp.1300664 15688089

[B67] Jongen P. J. Reiner W. O. Gabreëls F. J. (1980). Seven cases of Huntington's disease in childhood and levodopa induced improvement in the hypokinetic-rigid form. Clin. Neurol. Neurosurg. 82, 251–261 6165509 10.1016/0303-8467(80)90017-7

[B68] Julien C. L. Thompson J. C. Wild S. Yardumian P. Snowden J. S. Turner G. (2007). Psychiatric disorders in preclinical Huntington's disease. J. Neurol. Neurosurg. Psychiatry 78, 939–943 10.1136/jnnp.2006.103309 17178819 PMC2117854

[B69] Karishma K. K. Herbert J. (2002). Dehydroepiandrosterone (DHEA) stimulates neurogenesis in the hippocampus of the rat, promotes survival of newly formed neurons and prevents corticosterone-induced suppression. Eur. J. Neurosci. 16, 445–453 10.1046/j.1460-9568.2002.02099.x 12193187

[B70] Kingma E. M. van Duijn E. Timman R. van der Mast R. C. Roos R. A. C. (2008). Behavioral problems in Huntington's disease using the Problem Behaviors Assessment. Gen. Hosp. Psychiatry 30, 155–161 10.1016/j.genhosppsych.2007.11.005 18291297

[B71] Klempin F. Babu H. De Pietri Tonelli D. Alarcon E. Fabel K. Kempermann G. (2010). Oppositional effects of serotonin receptors 5-HT1a, 2, and 2c in the regulation of adult hippocampal neurogenesis. Front. Mol. Neurosci. 3:14 10.3389/fnmol.2010.00014 20721314 PMC2922940

[B72] Klivenyi P. Bende Z. Hartai Z. Penke Z. Nemeth H. Toldi J. (2006). Behaviour changes in a transgenic model of Huntington's disease. Behav. Brain Res. 169, 137–141 10.1016/j.bbr.2006.01.003 16443291

[B73] Kohl Z. Kandasamy M. Winner B. Aigner R. Gross C. Couillard-Despres S. (2007). Physical activity fails to rescue hippocampal neurogenesis deficits in the R6/2 mouse model of Huntington's disease. Brain Res. 1155, 24–33 10.1016/j.brainres.2007.04.039 17512917

[B74] Kornstein S. G. Schatzberg F. Thase M. E. Yonkers K. McCullough J. P. Keitner G. I. (2000). Gender differences in treatment response to sertraline versus imipramine in chronic depression. Am. J. Psychiatry 157, 1445–1452 10.1176/appi.ajp.157.9.1445 10964861

[B75] Krishnan V. Han M.-H. Graham D. L. Berton O. Renthal W. Russo S. J. (2007). Molecular adaptations underlying susceptibility and resistance to social defeat in brain reward regions. Cell 131, 391–404 10.1016/j.cell.2007.09.018 17956738

[B76] Krogias C. Strassburger K. Eyding J. Gold R. Norra C. Juckel G. (2011). Depression in patients with Huntington disease correlates with alterations of the brain stem raphe depicted by transcranial sonography. J. Psychiatry Neurosci. 36, 187–194 10.1503/jpn.100067 21138658 PMC3080514

[B77] Kupfer D. J. Frank E. Phillips M. L. (2012). Major depressive disorder: new clinical, neurobiological, and treatment perspectives. Lancet 379, 1045–1055 10.1016/S0140-6736(11)60602-8 22189047 PMC3397431

[B78] Landles C. Sathasivam K. Weiss A. Woodman B. Moffitt H. Finkbeiner S. (2010). Proteolysis of mutant huntingtin produces an exon 1 fragment that accumulates as an aggregated protein in neuronal nuclei in Huntington disease. J. Biol. Chem. 285, 8808–8823 10.1074/jbc.M109.075028 20086007 PMC2838303

[B79] Lazic S. E. Grote H. E. Armstrong R. J. E. Blakemore C. Hannan A. J. van Dellen A. (2004). Decreased hippocampal cell proliferation in R6/1 Huntington's mice. Neuroreport 15, 811–813 15073520 10.1097/00001756-200404090-00014

[B80] Lazic S. E. Grote H. E. Blakemore C. Hannan A. J. van Dellen A. Phillips W. (2006). Neurogenesis in the R6/1 transgenic mouse model of Huntington's disease: effects of environmental enrichment. Eur. J. Neurosci. 23, 1829–1838 10.1111/j.1460-9568.2006.04715.x 16623840

[B81] Lazo O. M. Gonzalez A. Ascano M. Kuruvilla R. Couve A. (2013). BDNF regulates Rab11-mediated recycling endosome dynamics to induce dendritic branching. J. Neurosci. 33, 6112–6122 .BDNF 10.1523/JNEUROSCI.4630-12.2013 23554492 PMC3684039

[B82] Leblhuber F. Peichl M. Neubauer C. Reisecker F. Steinparz F. X. Windhager E. (1995). Serum dehydroepiandrosterone and cortisol measurements in Huntington's chorea. J. Neurol. Sci. 132, 76–79 8523035 10.1016/0022-510x(95)00114-h

[B83] Lee J. Duan W. Mattson M. P. (2002). Evidence that brain-derived neurotrophic factor is required for basal neurogenesis and mediates, in part, the enhancement of neurogenesis by dietary restriction in the hippocampus of adult mice. J. Neurochem. 82, 1367–1375 10.1046/j.1471-4159.2002.01085.x 12354284

[B84] Levy M. L. Cummings J. L. Fairbanks L. A. Masterman D. Miller B. L. Craig A. H. (1998). Apathy is not depression. J. Neuropsy. Clin. Neurosci. 10, 314–319 9706539 10.1176/jnp.10.3.314

[B85] Li S. Gutekunst C. Hersch S. M. Li X. (1998). Interaction of Huntingtin-Associated Protein with Dynactin P150 Glued. J. Neurosci. 18, 1261–1269 9454836 10.1523/JNEUROSCI.18-04-01261.1998 PMC6792727

[B86] Li X. Valencia A. Sapp E. Masso N. Alexander J. Reeves P. (2010). Aberrant Rab11-Dependent trafficking of the neuronal glutamate transporter EAAC1 causes oxidative stress and cell death in Huntington's Disease. J. Neurosci. 30, 4552– 4561 10.1523/JNEUROSCI.5865-09.2010 20357106 PMC3842456

[B87] Li Y. Luikart B. W. Birnbaum S. Chen J. Kwon C.-H. Kernie S. G. (2008). TrkB regulates hippocampal neurogenesis and governs sensitivity to antidepressive treatment. Neuron 59, 399–412 10.1016/j.neuron.2008.06.023 18701066 PMC2655199

[B88] Liot G. Zala D. Pla P. Mottet G. Piel M. Saudou F. (2013). Mutant Huntingtin alters retrograde transport of TrkB receptors in striatal dendrites. J. Neurosci. 33, 6298–6309 10.1523/JNEUROSCI.2033-12.2013 23575829 PMC6619069

[B89] Lira A. Zhou M. Castanon N. Ansorge M. S. Gordon J. A. Francis J. H. (2003). Altered depression-related behaviors and functional changes in the dorsal raphe nucleus of serotonin transporter-deficient mice. Biol. Psychiatry 54, 960–971 10.1016/S0006-3223(03)00696-6 14625138

[B90] Luellen B. A. Bianco L. E. Schneider L. M. Andrews A. M. (2007). Reduced brain-derived neurotrophic factor is associated with a loss of serotonergic innervation in the hippocampus of aging mice. Genes. Brain. Behav. 6, 482–490 10.1111/j.1601-183X.2006.00279.x 17156118

[B91] Lyons W. E. Mamounas L. A. Ricaurte G. A. Coppola V. Reid S. W. Bora S. H. (1999). Brain-derived neurotrophic factor-deficient mice develop aggressiveness and hyperphagia in conjunction with brain serotonergic abnormalities. Proc. Natl. Acad. Sci. U.S.A. 96, 15239–15344 10611369 10.1073/pnas.96.26.15239 PMC24804

[B92] Madhav T. R. Pei Q. Zetterström T. S. (2001). Serotonergic cells of the rat raphe nuclei express mRNA of tyrosine kinase B (trkB), the high-affinity receptor for brain derived neurotrophic factor (BDNF). Mol. Brain Res. 93, 56–63 10.1016/S0169-328X(01)00183-8 11532338

[B93] Malberg J. E. Blendy J. A. (2005). Antidepressant action: to the nucleus and beyond. Trends Pharmacol. Sci. 26, 631–638 10.1016/j.tips.2005.10.005 16246434

[B94] Mangiarini L. Sathasivam K. Seller M. Cozens B. Harper A. Hetherington C. (1996). Exon 1 of the HD gene with an expanded CAG repeat is sufficient to cause a progressive neurological phenotype in transgenic mice. Cell 87, 493–506 8898202 10.1016/s0092-8674(00)81369-0

[B95] Marshall J. White K. Weaver M. Flury Wetherill L. Hui S. Stout J. C. (2007). Specific psychiatric manifestations among preclinical Huntington disease mutation carriers. Arch. Neurol. 64, 116–121 10.1001/archneur.64.1.116 17210818

[B96] Mayorga A. J. Dalvi A. Page M. E. Zimov-Levinson S. Hen R. (2001). Antidepressant-Like Behavioral Effects in 5HTR1A and 5HTR1B mutant mice. J. Pharmacol. Exp. Ther. 298, 1101–1107 11504807

[B97] McGuire J. R. Rong J. Li S.-H. Li X.-J. (2006). Interaction of Huntingtin-associated protein-1 with kinesin light chain: implications in intracellular trafficking in neurons. J. Biol. Chem. 281, 3552–3559 10.1074/jbc.M509806200 16339760

[B98] Menalled L. El-Khodor B. F. Patry M. Suárez-Fariñas M. Orenstein S. J. Zahasky B. (2009). Systematic behavioral evaluation of Huntington's disease transgenic and knock-in mouse models. Neurobiol. Dis. 35, 319–336 10.1016/j.nbd.2009.05.007 19464370 PMC2728344

[B99] Mendez-David I. David D. J. Darcet F. Wu V. W. Kerdine-Romer S. Gardier A. M. (2014). Rapid anxiolytic effect of 5-HT4 receptor agonist are mediated by a neurogenesis-independent mechanism. Neuropsychopharmacology 39, 1366–1378 10.1038/npp.2013.332 24287720 PMC3988540

[B100] Mestre T. A. Ferreira J. J. (2012). An evidence-based approach in the treatment of Huntington's disease. Parkinsonism Relat. Disord. 18, 316–320 10.1016/j.parkreldis.2011.10.021 22177624

[B101] Miller B. H. Schultz L. E. Gulati A. Su A. I. Pletcher M. T. (2010). Phenotypic characterization of a genetically diverse panel of mice for behavioral despair and anxiety. PLoS ONE 5:e14458 10.1371/journal.pone.0014458 21206921 PMC3012073

[B102] Milnerwood A. J. Raymond L. A. (2007). Corticostriatal synaptic function in mouse models of Huntington's disease: early effects of huntingtin repeat length and protein load. J. Physiol. 585, 817–831 10.1113/jphysiol.2007.142448 17947312 PMC2375504

[B103] Monteggia L. M. Barrot M. Powell C. M. Berton O. Galanis V. Gemelli T. (2004). Essential role of brain-derived neurotrophic factor in adult hippocampal function. Proc. Natl. Acad. Sci. U.S.A. 101, 10827–10832 10.1073/pnas.0402141101 15249684 PMC490019

[B104] Monteggia L. M. Luikart B. Barrot M. Theobold D. Malkovska I. Nef S. (2007). Brain-Derived neurotrophic factor conditional knockouts show gender differences in depression-related behaviors. Biol. Psychiatry 187–197 10.1016/j.biopsych.2006.03.021 16697351

[B105] Mozhui K. Karlsson R.-M. Kash T. L. Ihne J. Norcross M. Patel S. (2010). Strain differences in stress responsivity are associated with divergent amygdala gene expression and glutamate-mediated neuronal excitability. J. Neurosci. 30, 5357–5367 10.1523/JNEUROSCI.5017-09.2010 20392957 PMC2866495

[B106] Murray F. Smith D. W. Hutson P. H. (2008). Chronic low dose corticosterone exposure decreased hippocampal cell proliferation, volume and induced anxiety and depression like behaviors in mice. Eur. J. Pharmacol. 583, 115–127 10.1016/j.ejphar.2008.01.014 18289522

[B107] Nasir J. Floresco S. B. O'Kusky J. R. Diewert J. M. Richman J. Zeisler A. (1995). Targeted disruption of Huntington's disease results in embryonic lethality and behavioral and morphological consequences in heterozygotes. Cell 81, 811–823 7774020 10.1016/0092-8674(95)90542-1

[B108] Naver B. Stub C. Møller M. Fenger K. Hansen A. K. Hasholt L. (2003). Molecular and behavioral analysis of the r6/1 huntington's disease transgenic mouse. Neuroscience 122, 1049–1057 10.1016/j.neuroscience.2003.08.053 14643771

[B109] Nestler E. J. Barrot M. Dileone R. J. Eisch A. J. Gold S. J. Monteggia L. M. (2002). Neurobiology of depression review. Neuron 34, 13–25 10.1016/S0896-6273(02)00653-0 11931738

[B110] Nithianantharajah J. Barkus C. Murphy M. Hannan A. J. (2008). Gene-environment interactions modulating cognitive function and molecular correlates of synaptic plasticity in Huntington's disease transgenic mice. Neurobiol. Dis. 29, 490–504 10.1016/j.nbd.2007.11.006 18165017

[B111] Novak M. J. U. Tabrizi S. J. (2011). Huntington's disease: clinical presentation and treatment. Int. Rev. Neurobiol. 98, 297–323 10.1016/B978-0-12-381328-2.00013-4 21907093

[B112] Orvoen S. Pla P. Gardier A. M. Saudou F. David D. J. (2012). Huntington's disease knock-in male mice show specific anxiety-like behavior and altered neuronal maturation. Neurosci. Lett. 507, 127–132 10.1016/j.neulet.2011.11.063 22178857

[B113] Page M. E. Detke M. J. Dalvi A. Kirby L. G. Lucki I. (1999). Serotonergic mediation of the effects of fluoxetine, but not desipramine, in the rat forced swimming test. Psychopharmacology 147, 162–167 10591883 10.1007/s002130051156

[B114] Pang T. Y. C. Stam N. C. Nithianantharajah J. Howard M. L. Hannan A. J. (2006). Differential effects of voluntary physical exercise on behavioral and brain-derived neurotrophic factor expression deficits in Huntington's disease transgenic mice. Neuroscience 141, 569–584 10.1016/j.neuroscience.2006.04.013 16716524

[B115] Pang T. Y. Du X. Zajac M. S. Howard M. L. Hannan A. J. (2009). Altered serotonin receptor expression is associated with depression-related behavior in the R6/1 transgenic mouse model of Huntington's disease. Hum. Mol. Genet. 18, 753–766 10.1093/hmg/ddn385 19008301

[B116] Paulsen J. S. (2011). Cognitive impairment in huntington disease: diagnosis and treatment. Curr. Neurol. Neurosci. Rep. 11, 474–483 10.1007/s11910-011-0215-x 21861097 PMC3628771

[B117] Paulsen J. S. Nehl C. Hoth K. F. Kanz J. E. Benjamin M. Conybeare R. (2005). Depression and Stages of Huntington's Disease. J. Neuropsychiatr. 496–502 10.1176/appi.neuropsych.17.4.496 16387989

[B118] Peinemann A. Schuller S. Pohl C. Jahn T. Weindl A. Kassubek J. (2005). Executive dysfunction in early stages of Huntington's disease is associated with striatal and insular atrophy: a neuropsychological and voxel-based morphometric study. J. Neurol. Sci. 239, 11–19 10.1016/j.jns.2005.07.007 16185716

[B119] Peng Q. Masuda N. Jiang M. Li Q. Zhao M. Ross C. A. (2008). The antidepressant sertraline improves the phenotype, promotes neurogenesis and increases BDNF levels in the R6/2 Huntington's disease mouse model. Exp. Neurol. 210, 154–163 10.1016/j.expneurol.2007.10.015 18096160 PMC2278120

[B120] Phillips W. Morton A. J. Barker R. A. (2005). Abnormalities of neurogenesis in the R6/2 mouse model of Huntington's disease are attributable to the *in vivo* microenvironment. J. Neurosci. 25, 11564–11576 10.1523/JNEUROSCI.3796-05.2005 16354914 PMC6726042

[B121] Pineda J. R. Canals J. M. Bosch M. Adell A. Mengod G. Artigas F. (2005). Brain-derived neurotrophic factor modulate dopaminergic deficits in a transgenic mouse model of Huntington's disease. J. Neurochem. 93, 1057–1068 10.1111/j.1471-4159.2005.03047.x 15934928

[B122] Pla P. Orvoen S. Benstaali C. Dodier S. Gardier A. M. David D. J. (2013). Huntingtin acts non cell-autonomously on hippocampal neurogenesis and controls anxiety-related behaviors in adult mouse. PLoS ONE 8:e73902 10.1371/journal.pone.0073902 24019939 PMC3760801

[B123] Pouladi M. A. Graham R. K. Karasinska J. M. Xie Y. Santos R. D. Petersén A. (2009). Prevention of depressive behavior in the YAC128 mouse model of Huntington disease by mutation at residue 586 of huntingtin. Brain 132, 919–932 10.1093/brain/awp006 19224899

[B124] Pouladi M. A. Morton A. J. Hayden M. R. (2013). Choosing an animal model for the study of Huntington's disease. Nat. Rev. Neurosci. 14, 708–721 10.1038/nrn3570 24052178

[B125] Pouladi M. A. Stanek L. M. Xie Y. Franciosi S. Southwell A. L. Deng Y. (2012). Marked differences in neurochemistry and aggregates despite similar behavioral and neuropathological features of Huntington disease in the full-length BACHD and YAC128 mice. Hum. Mol. Genet. 21, 2219–2232 10.1093/hmg/dds037 22328089

[B126] Quesseveur G. David D. J. Gaillard M. C. Pla P. Wu M. V. Nguyen H. T. (2013). BDNF overexpression in mouse hippocampal astrocytes promotes local neurogenesis and elicits anxiolytic-like activities. Transl. Psychiatry 3, e253 10.1038/tp.2013.30 23632457 PMC3641417

[B127] Rainnie D. G. (1999). Serotonergic modulation of neurotransmission in the rat basolateral amygdala. J. Neurophysiol. 82, 69–85 10400936 10.1152/jn.1999.82.1.69

[B128] Rangone H. Poizat G. Troncoso J. Ross C. A. MacDonald M. E. Saudou F. (2004). The serum and glucocorticoid-induced kinase SGK inhibits mutant huntingtin-induced toxicity by phosphorylating serine 421 of huntingtin. Eur. J. Neurosci. 19, 273–279 10.1111/j.0953-816X.2003.03131.x 14725621

[B129] Rantamäki T. Hendolin P. Kankaanpää A. Mijatovic J. Piepponen P. Domenici E. (2007). Pharmacologically diverse antidepressants rapidly activate brain-derived neurotrophic factor receptor TrkB and induce phospholipase-Cgamma signaling pathways in mouse brain. Neuropsychopharmacology 32, 2152–2162 10.1038/sj.npp.1301345 17314919

[B130] Reedeker W. van der Mast R. C. Giltay E. J. Kooistra T. A. Roos R. A. van Duijn E. (2012). Psychiatric disorders in Huntington's disease: a 2-year follow-up study. Psychosomatics 53, 220–229 10.1016/j.psym.2011.12.010 22458993

[B131] Renoir T. Pang T. Y. C. Zajac M. S. Chan G. Du X. Leang L. (2012). Treatment of depressive-like behavior in Huntington's disease mice by chronic sertraline and exercise. Br. J. Pharmacol. 165, 1375–1389 10.1111/j.1476-5381.2011.01567.x 21718306 PMC3372723

[B132] Renoir T. Zajac M. S. Du X. Pang T. Y. Leang L. Chevarin C. (2011). Sexually dimorphic serotonergic dysfunction in a mouse model of Huntington's Disease and depression. PLoS ONE 6:e22133 10.1371/journal.pone.0022133 21760962 PMC3132782

[B133] Huntington Collaborative Research Group (1993). A novel gene containing a trinucleotide that is expanded and unstable on huntington's disease chromosomes. Cell 72, 971–983 8458085 10.1016/0092-8674(93)90585-e

[B134] Richards G. Messer J. Waldvogel H. J. Gibbons H. M. Dragunow M. Faull R. L. M. (2011). Up-regulation of the isoenzymes MAO-A and MAO-B in the human basal ganglia and pons in Huntington's disease revealed by quantitative enzyme radioautography. Brain Res. 1370, 204–214 10.1016/j.brainres.2010.11.020 21075085

[B135] Rong J. Li S. Sheng G. Wu M. Coblitz B. Li M. (2007). 14-3-3 protein interacts with Huntingtin-associated protein 1 and regulates its trafficking. J. Biol. Chem. 282, 4748–4756 10.1074/jbc.M609057200 17166838

[B136] Rosenblatt A. (2007). Neuropsychiatry of HD. Dialogues Clin. Neurosci. 9, 191–197 17726917 10.31887/DCNS.2007.9.2/arosenblatt PMC3181855

[B137] Rowe K. C. Paulsen J. S. Langbehn D. R. Wang C. Mills J. Beglinger L. J. (2012). Patterns of serotonergic antidepressant usage in prodromal Huntington disease. Psychiatry Res. 196, 309–314 10.1016/j.psychres.2011.09.005 22397915 PMC3763706

[B138] Rubinow D. R. Roca C. A. Schmidt P. J. Danaceau M. A. Putnam K. Cizza G. (2005). Testosterone suppression of CRH-stimulated cortisol in men. Neuropsychopharmacology 30, 1906–1912 10.1038/sj.npp.1300742 15841103 PMC1470424

[B139] Rudolph U. Möhler H. (2014). GABAA receptor subtypes: therapeutic potential in down syndrome, affective disorders, schizophrenia, and autism. Annu. Rev. Pharmacol. Toxicol. 54, 483–507 10.1146/annurev-pharmtox-011613-135947 24160694 PMC3997216

[B140] Saarelainen T. Hendolin P. Lucas G. Koponen E. Sairanen M. Macdonald E. (2003). Activation of the TrkB neurotrophin receptor is induced by antidepressant drugs and is required for antidepressant-induced behavioral effects. J. Neurosci. 23, 349–357 12514234 10.1523/JNEUROSCI.23-01-00349.2003 PMC6742146

[B141] Sairanen M. Lucas G. Ernfors P. Castrén M. Castrén E. (2005). Brain-derived neurotrophic factor and antidepressant drugs have different but coordinated effects on neuronal turnover, proliferation, and survival in the adult dentate gyrus. J. Neurosci. 25, 1089–1094 10.1523/JNEUROSCI.3741-04.2005 15689544 PMC6725966

[B142] Samuels B. Hen R. (2011). Neurogenesis and affective disorders. Eur. J. Neurosci. 33, 1152–1159 10.1111/j.1460-9568.2011.07614.x 21395859

[B143] Saydoff J. A. Garcia R. A. G. Browne S. E. Liu L. Sheng J. Brenneman D. Hu Z. (2006). Oral uridine pro-drug PN401 is neuroprotective in the R6/2 and N171-82Q mouse models of Huntington's disease. Neurobiol. Dis. 24, 455–465 10.1016/j.nbd.2006.08.011 17011205

[B144] Schilling G. Becher M. W. Sharp A. H. Jinnah H. A. Duan K. Kotzuk J. A. (1999). Intranuclear inclusions and neuritic aggregates in transgenic mice expressing a mutant N-terminal fragment of huntingtin. Hum. Mol. Genet. 8, 397–407 10.1093/hmg/8.3.397 9949199

[B145] Seo H. Sonntag K. C. Isacson O. (2004). Generalized brain and skin proteasome inhibition in Huntington's disease. Ann. Neurol. 56, 319–328 10.1002/ana.20207 15349858

[B146] Shirbin C. A. Chua P. Churchyard A. Lowndes G. Hannan A. J. Pang T. Y. (2013). Cortisol and depression in pre-diagnosed and early stage Huntington's disease. Psychoneuroendocrinology 38, 2439–2447 10.1016/j.psyneuen.2012.10.020 24074804

[B147] Simmons D. A. Rex C. S. Palmer L. Pandyarajan V. Fedulov V. Gall C. M. (2009). Up-regulating BDNF with an ampakine rescues synaptic plasticity and memory in Huntington's disease knockin mice. Proc. Natl. Acad. Sci. U.S.A. 106, 4906–4911 10.1073/pnas.0811228106 19264961 PMC2660722

[B148] Simpson J. M. Gil-Mohapel J. Pouladi M. A. Ghilan M. Xie Y. Hayden M. R. (2011). Altered adult hippocampal neurogenesis in the YAC128 transgenic mouse model of Huntington disease. Neurobiol. Dis. 41, 249–260 10.1016/j.nbd.2010.09.012 20875859

[B150] Smith M. M. Mills J. A. Epping E. A. Westervelt H. Paulsen J. S. PREDICT -HD Investigators of the Huntington Study Group. (2012). Depressive symptom severity is related to poorer cognitive performance in Prodromal Huntington Disease. Neuropsychology 26, 664–669 10.1037/a0029218 22846033 PMC3806339

[B151] Steward L. J. Bufton K. E. Hopkins P. C. Davies W. E. Barnes N. M. (1993). Reduced levels of 5-HT3 receptor recognition sites in the putamen of patients with Huntington's disease. Eur. J. Pharmacol. 242, 137–143 8253110 10.1016/0014-2999(93)90073-q

[B152] Strand A. D. Baguet Z. C. Aragaki A. K. Holmans P. Yang L. Cleren C. (2007). Expression profiling of Huntington's disease models suggests that brain-derived neurotrophic factor depletion plays a major role in striatal degeneration. J. Neurosci. 27, 11758–11768 10.1523/JNEUROSCI.2461-07.2007 17959817 PMC6673215

[B153] Sullivan F. R. Bird E. D. Alpay M. Cha J. H. (2001). Remotivation therapy and Huntington's disease. J. Neurosci. Nurs. 33, 136–142 11413658 10.1097/01376517-200106000-00005

[B154] Thompson J. C. Harris J. Sollom A. C. Stopford C. L. Howard E. Snowden J. S. (2012). Longitudinal evaluation of neuropsychiatric symptoms in Huntington's disease. J. Neuropsychiatry Clin. Neurosci. 24, 53–60 10.1176/appi.neuropsych.11030057 22450614

[B156] Trivedi M. H. Greer T. L. (2014). Cognitive dysfunction in unipolar depression: implications for treatment. J. Affect. Dis. 152, 19–27 10.1016/j.jad.2013.09.012 24215896

[B157] Twelvetrees A. E. Yuen E. Y. Arancibia-Carcamo I. L. MacAskill A. F. Rostaing P. Lumb M. J. (2010). Delivery of GABAARs to synapses is mediated by HAP1-KIF5 and disrupted by mutant huntingtin. Neuron 65, 53–65 10.1016/j.neuron.2009.12.007 20152113 PMC2841506

[B158] Tyler W. J. Pozzo-Miller L. (2003). Miniature synaptic transmission and BDNF modulate dendritic spine growth and form in rat CA1 neurones. J. Physiol. 553, 497–509 10.1113/jphysiol.2003.052639 14500767 PMC2343578

[B159] Van Duijn E. Kingma E. M. Timman R. Zitman F. G. Tibben A. Roos R. A. C. (2008). Cross-sectional study on prevalences of psychiatric disorders in mutation carriers of Huntington's disease compared with mutation-negative first degree relatives. J. Clin. Psychiatry 69, 1804–1810 19026253 10.4088/jcp.v69n1116

[B160] Van Duijn E. Selis M. A. Giltay E. J. Zitman F. G. Roos R. A. C. van Pelt H. (2010). Hypothalamic-pituitary-adrenal axis functioning in Huntington's disease mutation carriers compared with mutation-negative first-degree controls. Brain Res. Bull. 83, 232–237 10.1016/j.brainresbull.2010.08.006 20713132

[B162] Von Bohlen und Halbach O. Krause S. Medina D. Sciarretta C. Minichiello L. Unsicker K. (2006). Regional- and age-dependent reduction in trkB receptor expression in the hippocampus is associated with altered spine morphologies. Biol. Psychiatry 59, 793–800 10.1016/j.biopsych.2005.08.025 16325153

[B163] Von Bohlen und Halbach O. Minichiello L. Unsicker K. (2008). TrkB but not trkC receptors are necessary for postnatal maintenance of hippocampal spines. Neurobiol. Aging 29, 1247–1255 10.1016/j.neurobiolaging.2007.02.028 17442456

[B164] Waeber C. Palacios J. M. (1989). Serotonin-1 receptor binding sites in the human basal ganglia are decreased in Huntington's chorea but not in Parkinson's disease: a quantitative *in vitro* autoradiography study. Neuroscience 32, 337–347 2531301 10.1016/0306-4522(89)90082-1

[B165] Walker F. O. (2007). Huntington's disease. Lancet 369, 218–228 10.1016/S0140-6736(07)60111-1 17240289

[B166] Walker T. L. Turnbull G. W. Mackay E. W. Hannan A. J. Bartlett P. F. (2011). The latent stem cell population is retained in the hippocampus of transgenic Huntington's disease mice but not wild-type mice. PLoS ONE 6:e18153 10.1371/journal.pone.0018153 21455316 PMC3063801

[B167] Wang H. Chen X. Li Y. Tang T.-S. Bezprozvanny I. (2010). Tetrabenazine is neuroprotective in Huntington's disease mice. Mol. Neurodegener. 5, 18 10.1186/1750-1326-5-18 20420689 PMC2873255

[B168] Wong E. H. Reynolds G. P. Bonhaus D. W. Hsu S. Eglen R. M. (1996). Characterization of 3HGR 113808 binding to 5-HT4 receptors in brain tissues from patients with neurodegenerative disorders. Behav. Brain Res. 73, 249–252 8788512 10.1016/0166-4328(96)00106-4

[B169] Xia L. Deloménie C. David I. Rainer Q. Marouard M. Delacroix H. (2012). Ventral hippocampal molecular pathways and impaired neurogenesis associated with 5-HT 1A and 5-HT 1B receptors disruption in mice. Neurosci. Lett. 521, 20–25 10.1016/j.neulet.2012.05.046 22622174

[B170] Xie Y. Hayden M. R. Xu B. (2010). BDNF overexpression in the forebrain rescues Huntington's disease phenotypes in YAC128 mice. J. Neurosci. 30, 14708–14718 10.1523/JNEUROSCI.1637-10.2010 21048129 PMC2989389

[B171] Yohrling G. J. IV. Jiang G. C. T. DeJohn M. M. Robertson D. J. Vrana K. E. Cha J. H. J. (2002). Inhibition of tryptophan hydroxylase activity and decreased 5-HT 1A receptor binding in a mouse model of Huntington's disease. J. Neurochem. 82, 1416–1423 10.1046/j.1471-4159.2002.01084.x 12354289

[B172] Young A. B. Greenamyre J. T. Hollingsworth Z. Albin R. D'Amato C. Shoulson I. R. A. (1988). NMDA Receptor Losses in Putamen from Patients with Huntington's Disease. Science 241, 981–983 2841762 10.1126/science.2841762

[B173] Yu H. Chen Z. (2011). The role of BDNF in depression on the basis of its location in the neural circuitry. Acta Pharmacol. Sin. 32, 3–11 10.1038/aps.2010.184 21131999 PMC4003317

[B174] Yuen E. Y. Wei J. Zhong P. Yan Z. (2012). Disrupted GABAAR trafficking and synaptic inhibition in a mouse model of Huntington's disease. Neurobiol. Dis. 46, 497–502 10.1016/j.nbd.2012.02.015 22402331 PMC3323696

[B175] Zajac M. S. Pang T. Y. C. Wong N. Weinrich B. Leang L. S. K. Craig J. M. (2010). Wheel running and environmental enrichment differentially modify exon-specific BDNF expression in the hippocampus of wild-type and pre-motor symptomatic male and female Huntington's disease mice. Hippocampus 20, 621–636 10.1002/hipo.20658 19499586

[B176] Zala D. Colin E. Rangone H. Liot G. Humbert S. Saudou F. (2008). Phosphorylation of mutant huntingtin at S421 restores anterograde and retrograde transport in neurons. Hum. Mol. Genet. 17, 3837–3846 10.1093/hmg/ddn281 18772195

[B177] Zeitlin S. Liu J. P. Chapman D. L. Papaioannou V. E. Efstratiadis A. (1995). Increased apoptosis and early embryonic lethality in mice nullizygous for the Huntington's disease gene homologue. Nat. Genet. 11, 155–163 7550343 10.1038/ng1095-155

[B184] Zuccato C. Ciammola A. Rigamonti D. Leavitt B. R. Goffredo D. Conti L. (2001). Loss of huntingtin-mediated BDNF gene transcription in Huntington's disease. Science 293, 493–498 10.1126/science.1059581 11408619

[B178] Zuccato C. Liber D. Ramos C. Tarditi A. Rigamonti D. Tartari M. (2005). Progressive loss of BDNF in a mouse model of Huntington's disease and rescue by BDNF delivery. Pharmacol. Res. 52, 133–139 10.1016/j.phrs.2005.01.001 15967378

[B179] Zuccato C. Marullo M. Conforti P. MacDonald M. E. Tartari M. Cattaneo E. (2008). Systematic assessment of BDNF and its receptor levels in human cortices affected by Huntington's disease. Brain Pathol. 18, 225–238 10.1111/j.1750-3639.2007.00111.x 18093249 PMC8095509

[B180] Zuccato C. Tartari M. Crotti A. Goffredo D. Valenza M. Conti L. (2003). Huntingtin interacts with REST/NRSF to modulate the transcription of NRSE-controlled neuronal genes. Nat. Genet. 35, 76–83 10.1038/ng1219 12881722

[B181] Zuccato C. Valenza M. Cattaneo E. (2010). Molecular Mechanisms and Potential Therapeutical Targets in Huntington's Disease. Physiol. Rev. 905–981 10.1152/physrev.00041.2009 20664076

